# Supplementary material for: Modular assembly of the principal microtubule nucleator γ-TuRC
Source: Nat Commun. 2022 Jan 25;13:473. doi: 10.1038/s41467-022-28079-0 (PMC8789826; doi:10.1038/s41467-022-28079-0)

Supplementary Information

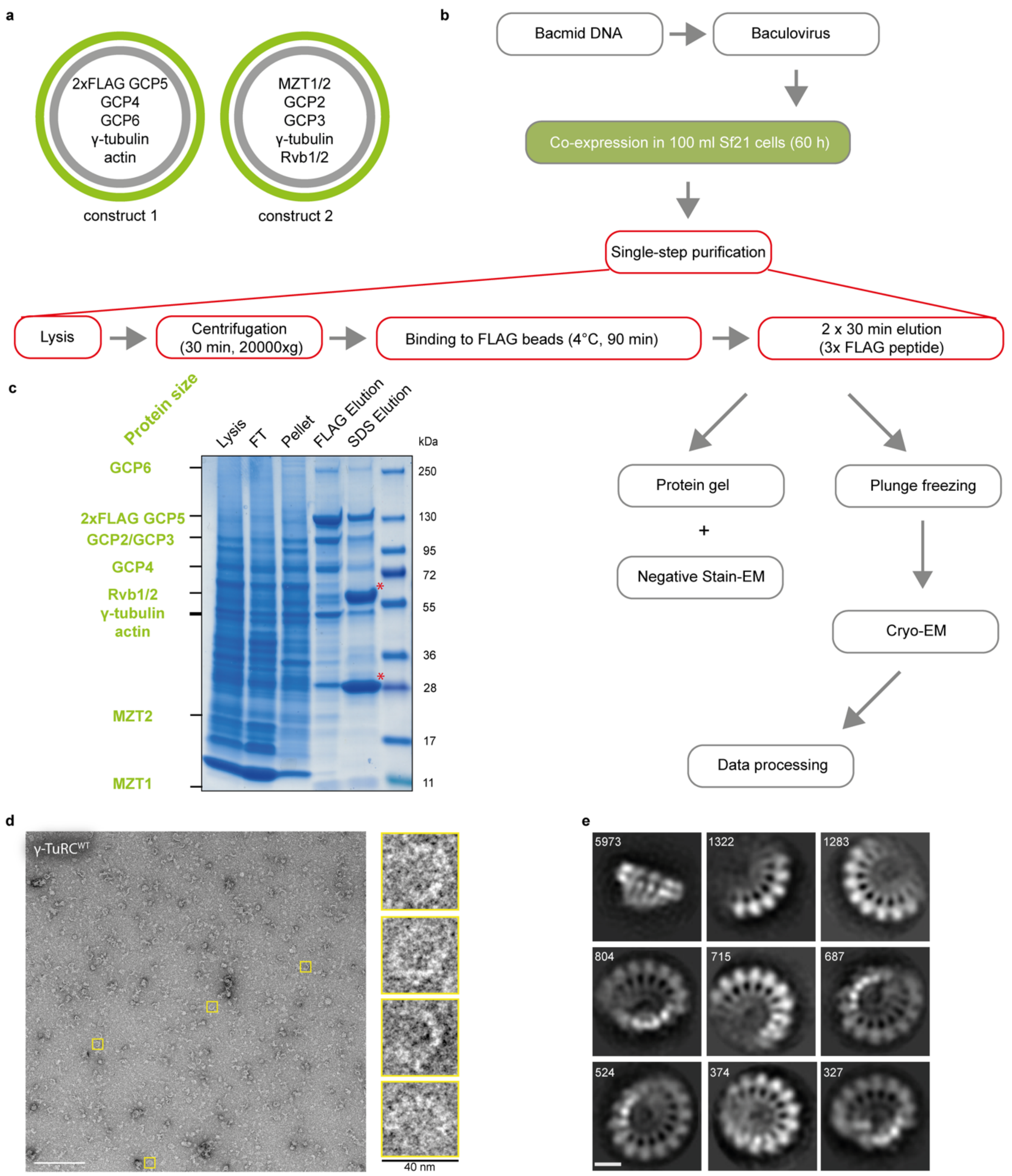

Supplementary Fig. 1: Purification and initial characterization of recombinant human γ-TuRC. a Cloned MultiBac constructs (construct 1 and 2) with the genes of human γ-TuRC (see methods). **b** Experimental pipeline of the recombinant γ-TuRC experiments. Baculoviruses of each construct were used to co-express all eleven genes in Sf21 insect cells. Cell pellets from 100 ml cells (green box) were used for single step FLAG purification against 2xFLAG GCP5 (red boxes). Flag elutions were screened via SDS-PAGE (**c**) and negative stain EM (**d**,**e**), and used for plunge freezing and subsequent cryo-EM analysis. **c** Coomassie-stained SDS-PAGE of the γ-TuRC purification sample used for cryo-EM showing the cell lysate (lysis), cell pellet (pellet), flow through (FT), and several proteins co-purified with 2xFLAG GCP5 in the FLAG elution. Expected protein sizes are indicated (green). FLAG beads were boiled with SDS loading buffer after FLAG peptide elution; red asterisks indicate heavy and light chain of FLAG antibodies. **d** Representative negative stain EM micrograph of the FLAG elution shown in (**c**), scale bar 250

nm**,** individual particles are highlighted in yellow boxes (40 nm). **e** Representative negative stain EM 2D class averages from the γ-TuRC sample used for cryo-EM analysis. Particle numbers are given in the left upper corner of the images. Scale bar: 10 nm. Data shown in **c**-**e** are from one experiment with no repetition of SDS-page and negative stain EM imaging. Purifications were repeated more than n=3 times.

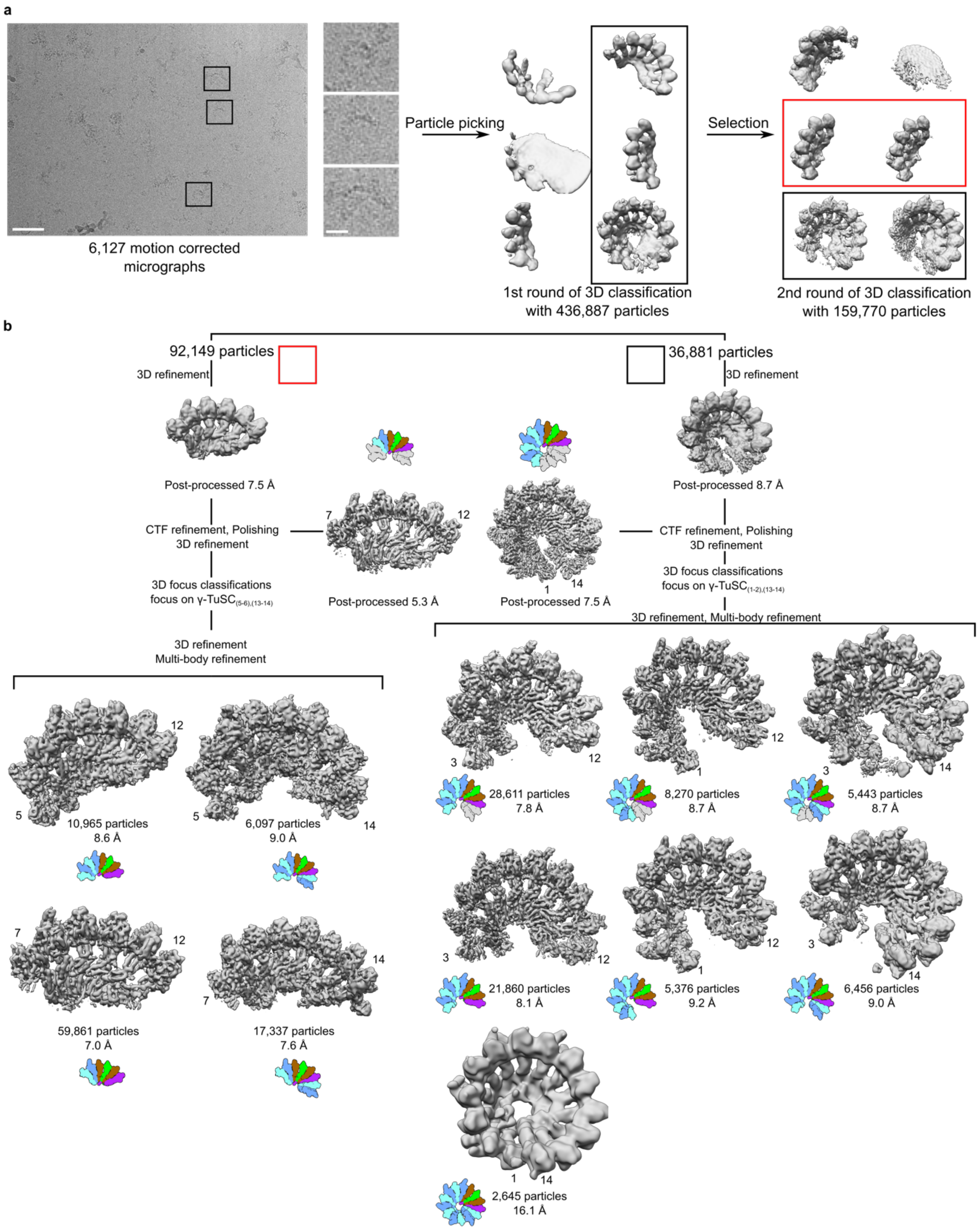

Supplementary Fig. 2: Processing scheme for the recombinant human γ-TuRC. a Zoom on a representative micrograph and three representative particles. Scale bars correspond to 80 nm (micrograph) and 20 nm (particles), respectively. Two initial rounds of 3D classification separated false positive particles from large and small γ-TuRC assemblies. Number of input particles for both classification rounds is indicated. Classes highlighted in red and black contain the sets of particles further processed in the left and right branch of the processing scheme in (**b**), respectively. **b** Processing of selected particles from (**a**) in two separate branches. Number of particles and resolution for each 3D reconstruction is indicated. The first and the last spokes visible in the reconstructions are numbered. Initially, particles in both branches were subjected to CTF refinement, Bayesian polishing and 3D autorefinement. Several consecutive rounds of focused 3D classification led to subsets of the particles corresponding to the different assembly states. For each reconstruction, the small icon indicates the set of particles

used during 3D refinement. Stoichiometric spokes are colored as in Fig. 1a. Substoichiometric spokes are depicted in grey. Spokes not shown in the icon are completely absent in the reconstruction. Data shown in **a** are from one acquisition with no repetition. Source data are provided as a Source Data file.

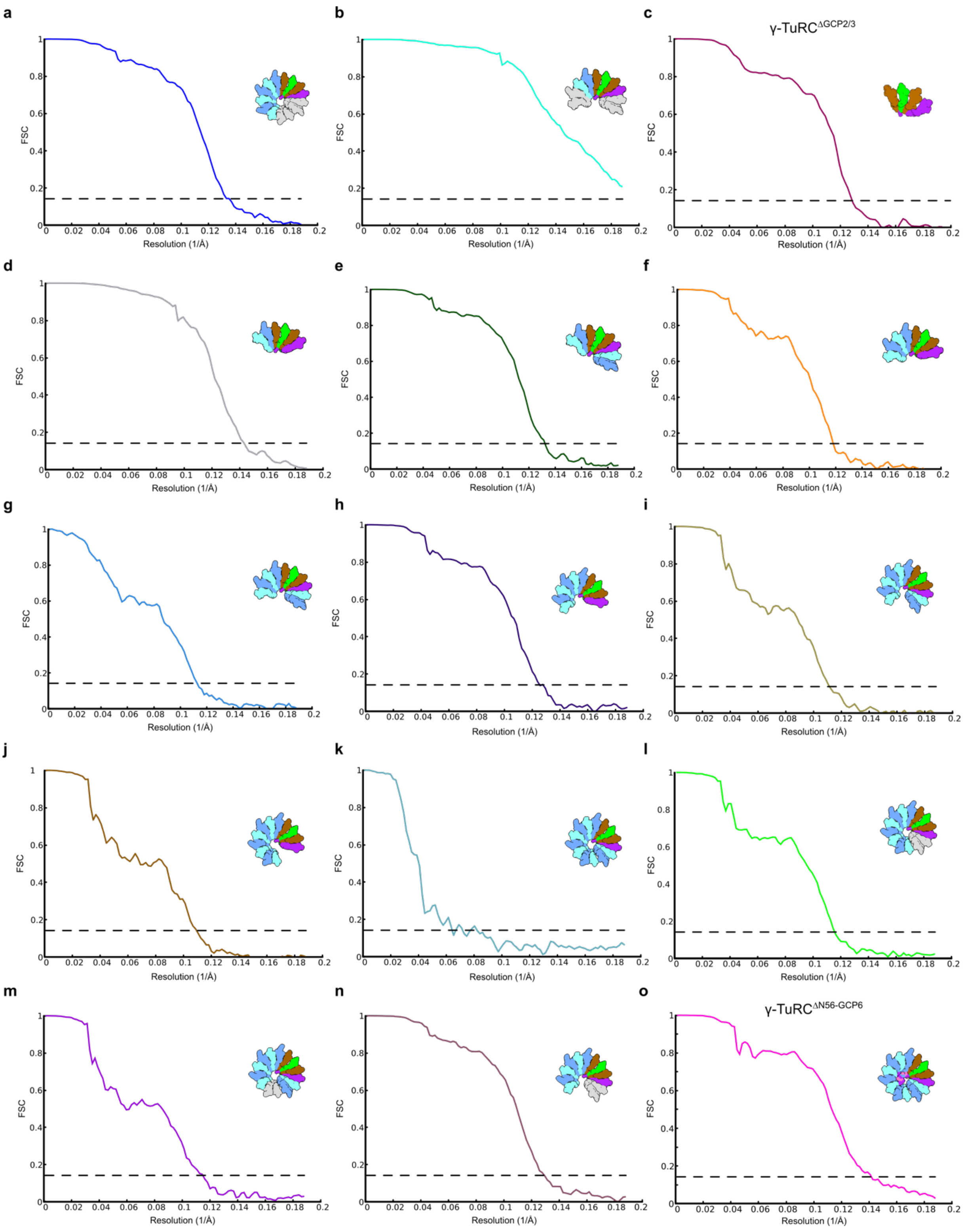

Supplementary Fig. 3: Fourier shell correlation (FSC) plots of all cryo-EM reconstructions described in the manuscript. **a** FSC curve for recombinant wild-type γ-TuRC (spokes 1-14). **b** FSC curve recombinant wild-type γ-TuRC, 6-spoked assembly intermediate (spokes 7-12). **c** FSC curve for recombinant γ-TuRC$^{\Delta GCP2/3}$. **d** FSC curve for recombinant wild-type γ-TuRC, 6-spoked assembly intermediate (spokes 7-12, homogeneous dataset). **e** FSC curve for recombinant wild-type γ-TuRC, 8-spoked assembly intermediate (spokes 7-14). **f** FSC curve for recombinant wild-type γ-TuRC, 8-spoked assembly intermediate (spokes 5-12). **g** FSC curve for recombinant wild-type γ-TuRC, 10-spoked assembly intermediate (spokes 5-14). **h** FSC curve for recombinant wild-type γ-TuRC, 10-

spoked assembly intermediate (spokes 3-12, homogeneous dataset). **i** FSC curve for recombinant wild-type γ-TuRC, 12-spoked assembly intermediate (spokes 3-14, homogeneous dataset). **j** FSC curve for recombinant wild-type γ-TuRC, 12-spoked assembly intermediate (spokes 1-12, homogeneous dataset). **k** FSC curve for recombinant wild-type γ-TuRC (spokes 1-14, homogeneous dataset). **l** FSC curve for recombinant wild-type γ-TuRC, 12-spoked assembly intermediate (spokes 1-12 substoichiometric spokes 13-14). **m** FSC curve for recombinant wild-type γ-TuRC, 12-spoked assembly intermediate (spokes 3-14 substoichiometric spokes 1-2). **n** FSC curve for recombinant wild-type γ-TuRC, 10-spoked assembly intermediate (spokes 3-12, substoichiometric spokes 13-14). **o** FSC curve for recombinant γ-TuRC$^{\Delta N56\text{-}GCP6}$. For recombinant wild-type γ-TuRC, the set of particles used for each reconstruction is also indicated by icons as in Supplementary Fig. 2. The FSC gold standard threshold of 0.143 is indicated by a dashed line in all plots. All plots were prepared in Gnuplot v5.2[1]. Source data are provided as a Source Data file.

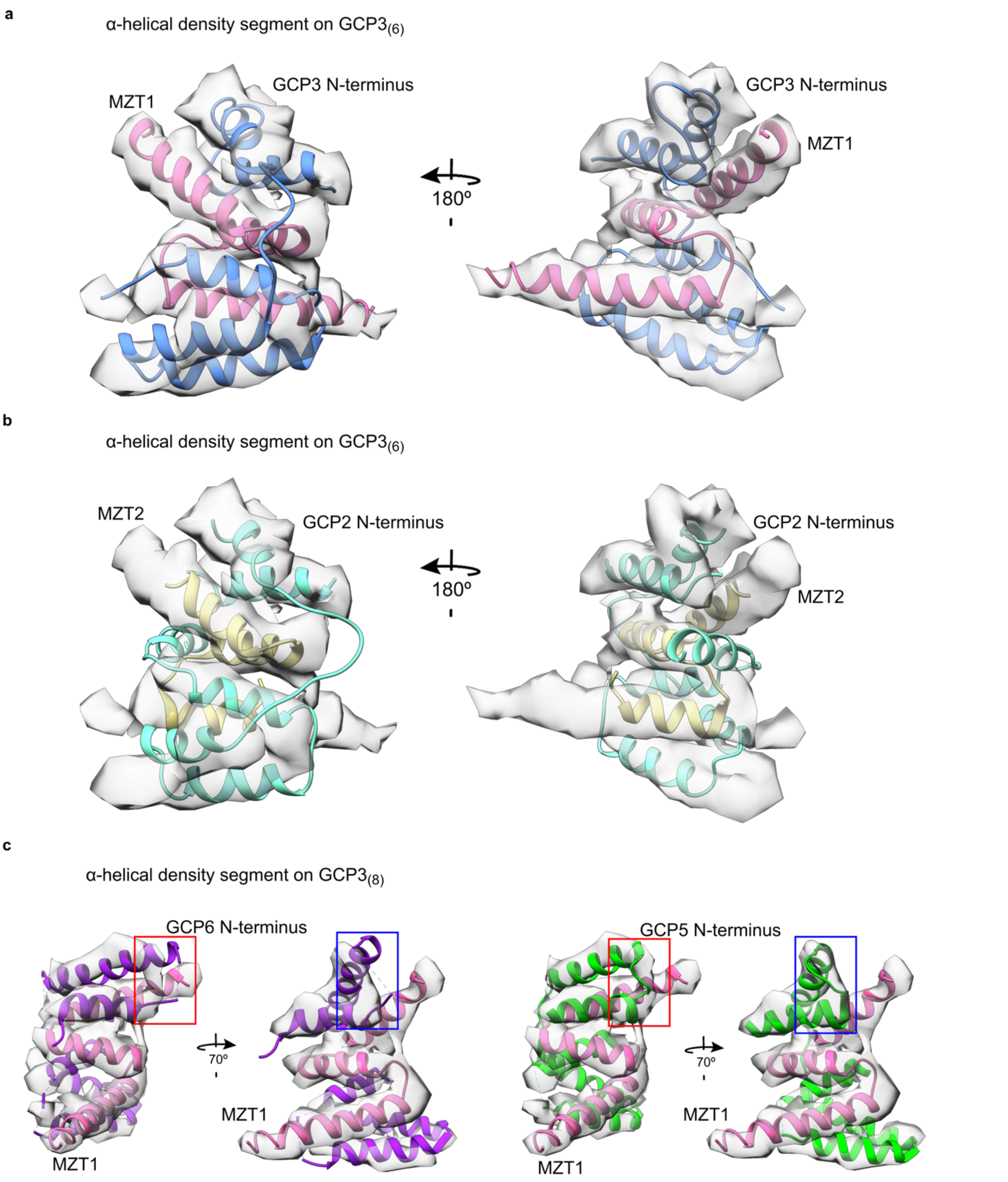

Supplementary Fig. 4: The α-helical density segments on the outer surface of the γ-TuRC represent MZT1-GCP modules. a Rigid body fit of the MZT1 (pink) - GCP3 (blue) module (PDB 6X0U) into the α-helical density segment associated with GCP3$_{(6)}$ of the γ-TuRC reconstruction. The model excellently explains the density segment. The GCP3$_{(6)}$ density segment is representative for the density segments on GCP3$_{(2)}$, GCP3$_{(4)}$ and GCP3$_{(14)}$. **b** Rigid body fit of the MZT2 (khaki) - GCP2 (cyan) module (PDB 6X0V) into the same density segment. Based on the length and number of resolved α-helices, we can exclude that the density segments correspond to MZT2-GCP modules. **c** Rigid body fit of MZT1 (pink) in complex with the GCP6 N-terminus (purple) (PDB 6M33) or the GCP5 N-terminus (green) (PDB 6L81) into the α-helical density segment associated with GCP3$_{(8)}$ of the 6-spoke intermediate. Red and blue boxes highlight density segments important for identification of the GCP variant.

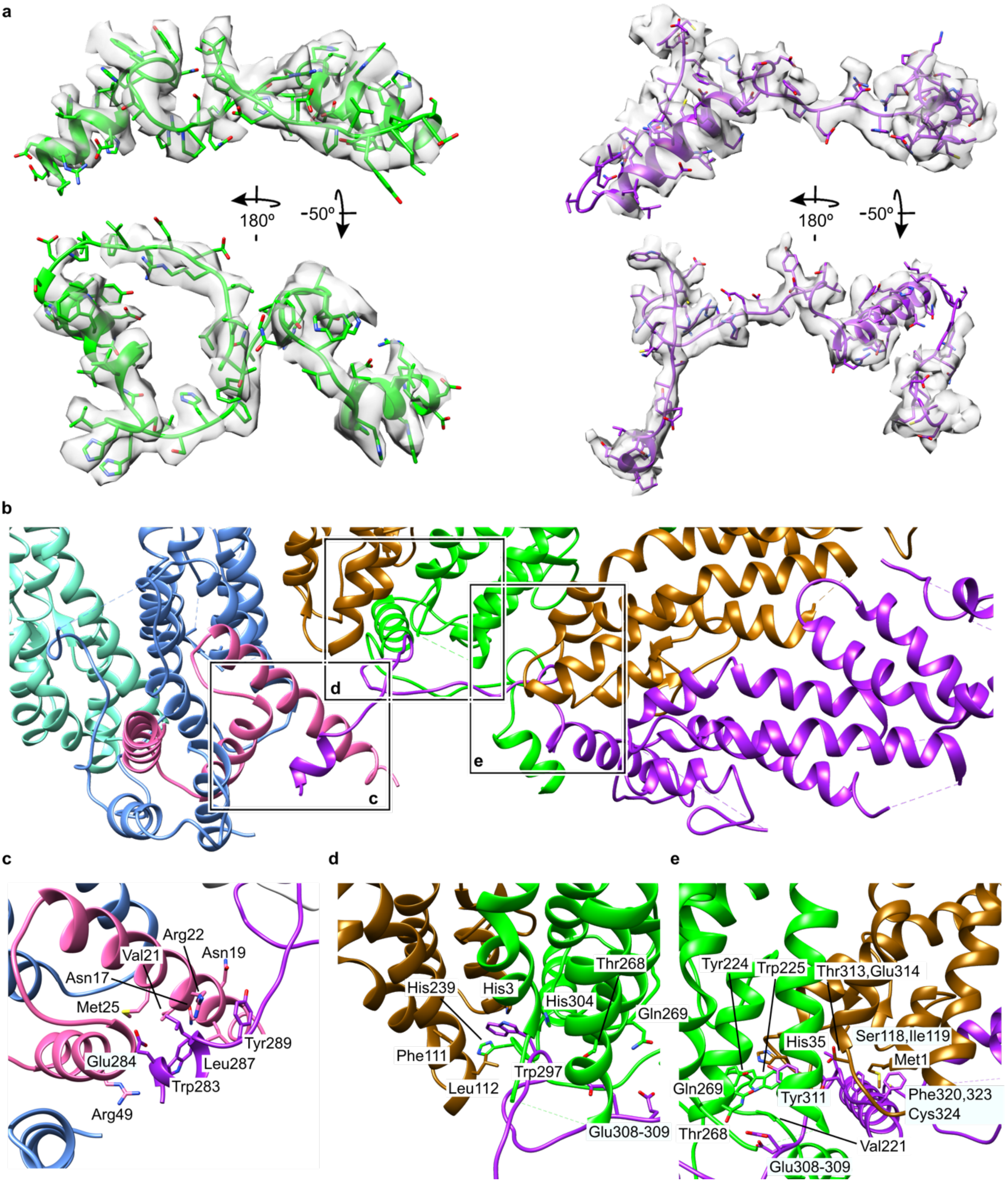

Supplementary Fig. 5: The GCP6 GRIP1-NTE stabilizes the 6-spoke intermediate. **a** N-terminal segments of GCP5 (210-266) and GCP6 (282-351) were newly built into published cryo-EM reconstructions (EMD-21074, EMD-21069)[2]. Atomic models superposed to the respective density segments. **b** Atomic model for the 6-spoke intermediate including the newly built segments of GCP5 and GCP6. Zoom on the GRIP1 domains and N-terminal extensions. Coloring as in Fig. 1a. Boxes indicate the position of zoomed views in panels (**c-e**). **c-e** Zoom on the interaction areas between the GCP6 GRIP1-NTE and MZT1 (**c**), $GCP4_{(9)}$ and $GCP5_{(10)}$ (**d**) and $GCP5_{(10)}$ and $GCP4_{(11)}$ (**e**) Side chains of residues involved in interactions are shown and labelled.

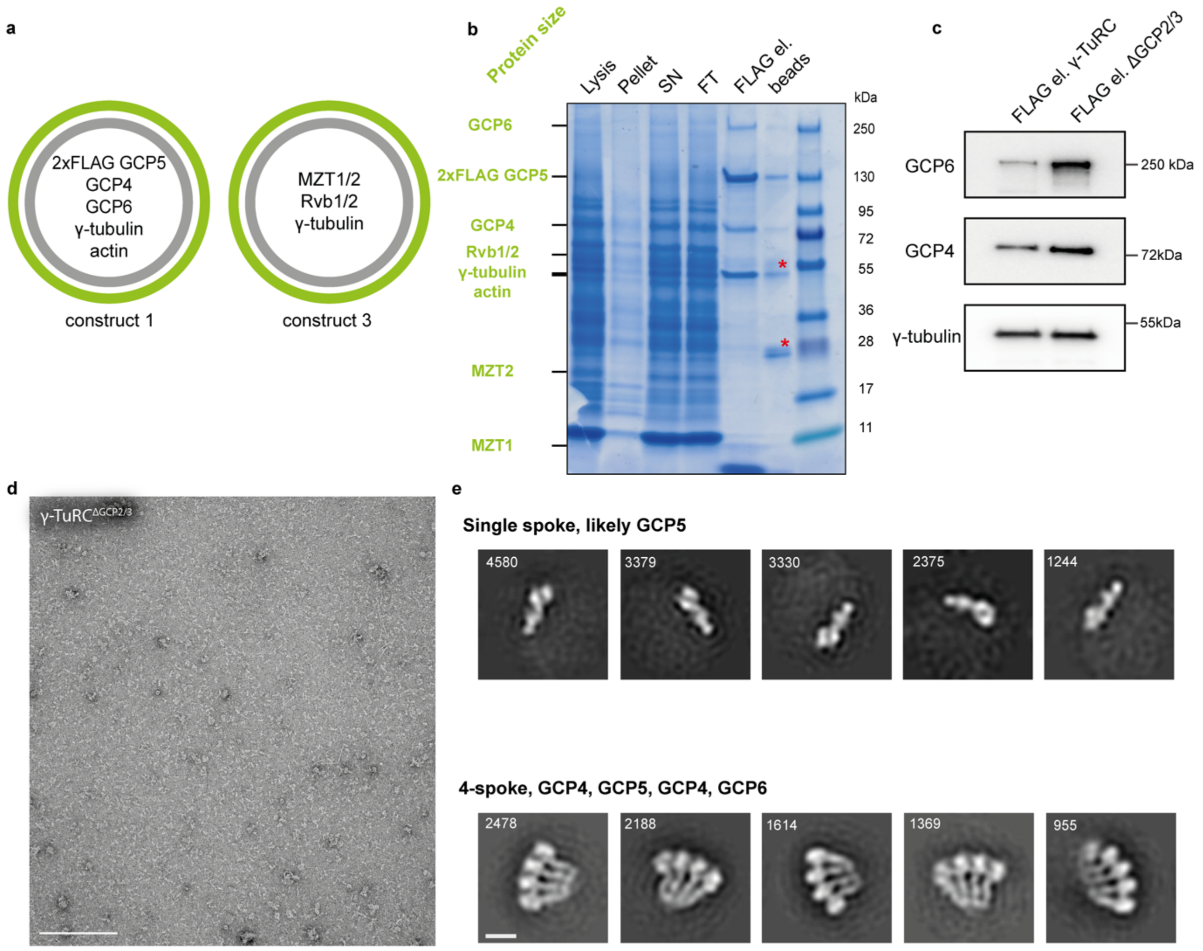

Supplementary Fig. 6: GCP4-6 can form a 4-spoke assembly when GCP2 and GCP3 are omitted from the expression system. a Cloned MultiBac constructs (construct 1 and construct 3) with the genes of human γ-TuRC components, omitting GCP2/3 co-expression (γ-TuRC$^{\Delta GCP2/3}$, see methods). Protein expression and purification was performed as described for wild-type γ-TuRC samples (Supplementary Fig. 1b). **b** Coomassie stained SDS-PAGE of complex purification showing the cell lysate (lysis), cell pellet (pellet), supernatant (SN), flowthrough (FT), and proteins copurified with 2xFLAG GCP5 in the FLAG elution. Expected protein sizes are indicated (green). FLAG beads were boiled with SDS loading buffer after FLAG peptide elution, red stars indicate heavy and light chain of FLAG antibodies. **c** Sections of immunoblot analysis of FLAG elutions (FLAG el.) from wild-type γ-TuRC (Supplementary Fig. 1) and ΔGCP2/3, against GCP6, GCP4 and γ-tubulin antibodies. **d** Representative negative stain EM micrograph of the FLAG elution in (**b**), scale bar 250 nm. **e** Representative negative stain EM 2D class averages. 2D classification resulted in two major particle populations: single spokes (likely 2xFLAG-GCP5), and 4-spoked subcomplexes corresponding to GCP4-5-4-6. Particle numbers are given in the left upper corner of the images. Scale bar: 10 nm. Data shown in **b-e** are from one experiment with no repetition of SDS-page and negative stain EM imaging and one repetition of immunoblot analysis. Protein complex expression and purification were repeated n=3 times.

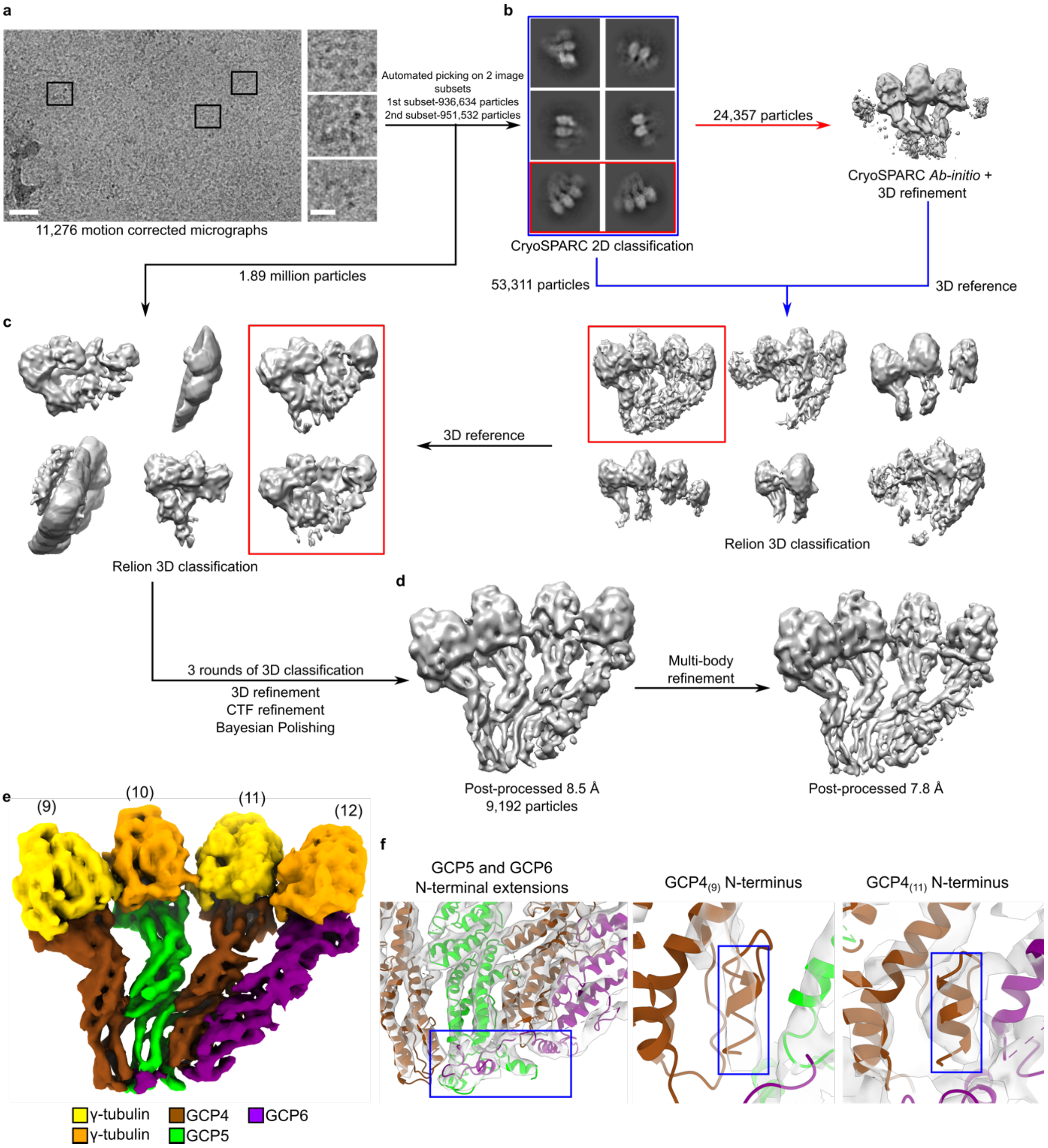

Supplementary Fig. 7: Cryo-EM data processing and density analysis of recombinant human γ-TuRC$^{\Delta GCP2/3}$. **a** Zoom on a representative micrograph and three representative particles. Scale bars correspond to 30 nm (micrograph) and 10 nm (particles), respectively. **b** Auto-picked particles were subjected to 2D classification in CryoSPARC. Six representative classes are shown. The highlighted 2D classes (red box) were used for *ab-initio* reconstruction and homogeneous refinement in CryoSPARC to generate an initial 3D reference. The density from (**a**) was used for 3D classification of an extended set of particles (blue box) in Relion to generate a more refined 3D reference, highlighted in red among the resultant 3D classes. **c** All picked particles were divided into subsets and subjected to several tiers of 3D classification using the reference from (**b**). 3D Classes after the 1st round of 3D classification are shown and selected 3D classes are highlighted. Three additional rounds of 3D classification resulted in the final set of particles, which were subjected to 3D refinement, CTF refinement and Bayesian particle polishing. **d** The final polished set of particles was subjected to 3D refinement (left) and multi-body refinement (right). **e** Cryo-EM density of the 4-spoke intermediate after multi-body refinement at 7.8 Å resolution. Colouring as indicated. Spoke numbering according to fully assembled γ-TuRC. **f** Zoom on density segments and atomic models

of the GCP N-termini in the 4-spoke intermediate. Left, N-terminal extension of GCP5 (green model) and GCP6 (purple model). Right, Last N-terminal helix in $GCP4_{(9)}$ and $GCP4_{(11)}$ (brown model). N-termini are highlighted in blue boxes. Data shown in **a** are from one acquisition with no repetition.

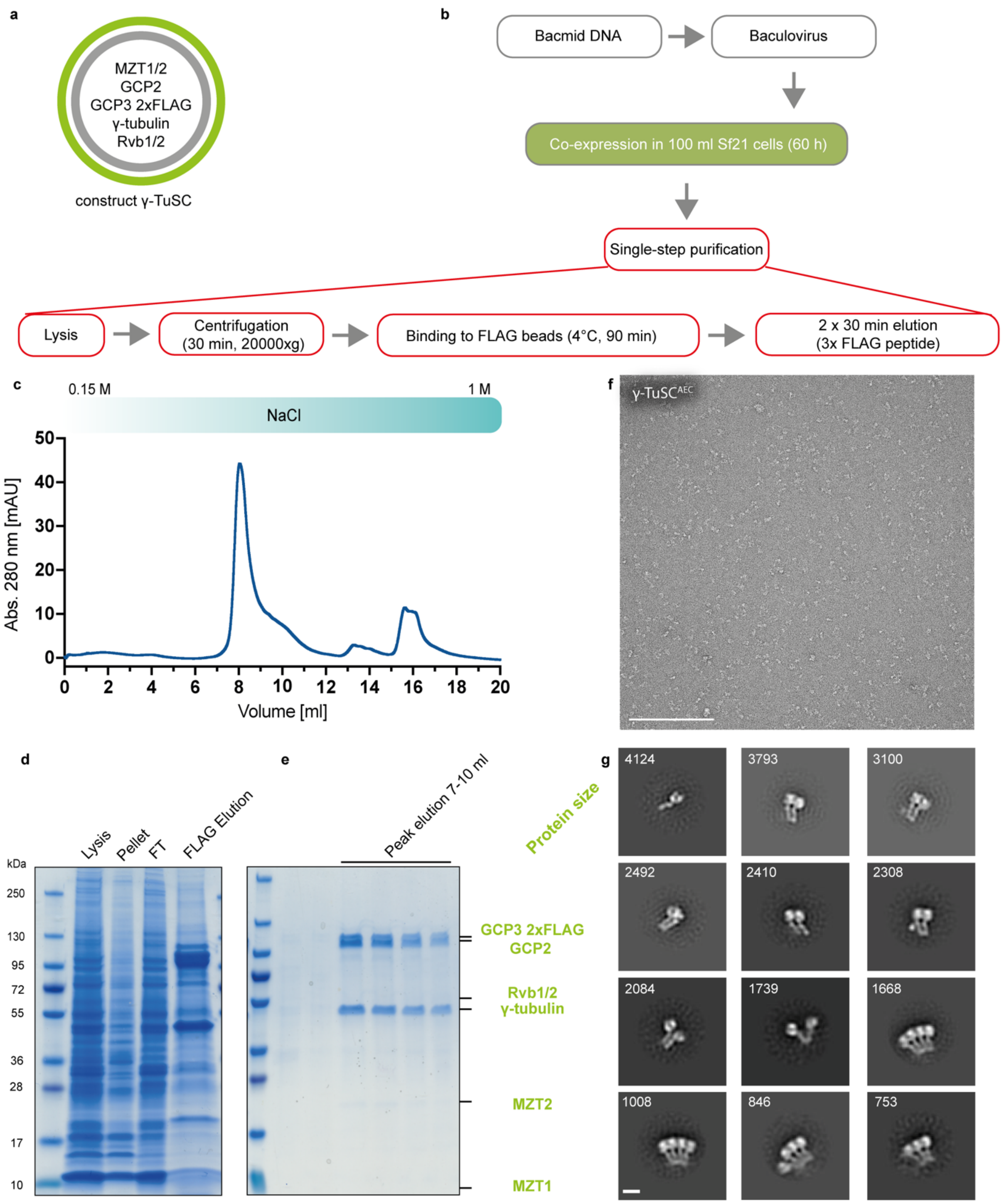

Supplementary Fig. 8: Recombinant expression and purification of human γ-TuSC. A Cloned MultiBac construct (construct γ-TuSC) with the genes of human γ-TuSC and the RUVBL1-RUVBL2 ATPase (Rvb1/2, see methods). **b** Experimental pipeline of the recombinant γ-TuSC purification. Baculoviruses of the construct were used to co-express the 7 genes in Sf21 insect cells. Cell pellets from 100 ml cells (green box) were used for single step FLAG purification against GCP3-2xFLAG (red boxes). FLAG elution was further purified via anion exchange chromatography (AEC) using a MonoQ (5/50 GL, Cytiva) column. **c** Chromatogram of AEC purification of recombinant γ-TuSC. **d,e** Coomassie stained SDS-PAGE of γ-TuSC purifications showing the cell lysate (lysis), cell pellet (Pellet), flowthrough (FT), and several proteins co-purified with GCP3 2xFLAG in the FLAG elution (**d**), and peak fraction of AEC run (**e**). Expected protein sizes are indicated (green). **f** Representative negative stain EM micrograph of γ-TuSC after AEC, scale bar 250 nm. **g** Representative negative stain EM 2D class averages. Particle

numbers are given in the left upper corner of the images. Scale bar: 10 nm. Data shown in **c**-**g** are from one experiment with no repetition of SDS-page and negative stain EM imaging. Protein complex expression and purification were repeated n=3 times. Source data are provided as a Source Data file.

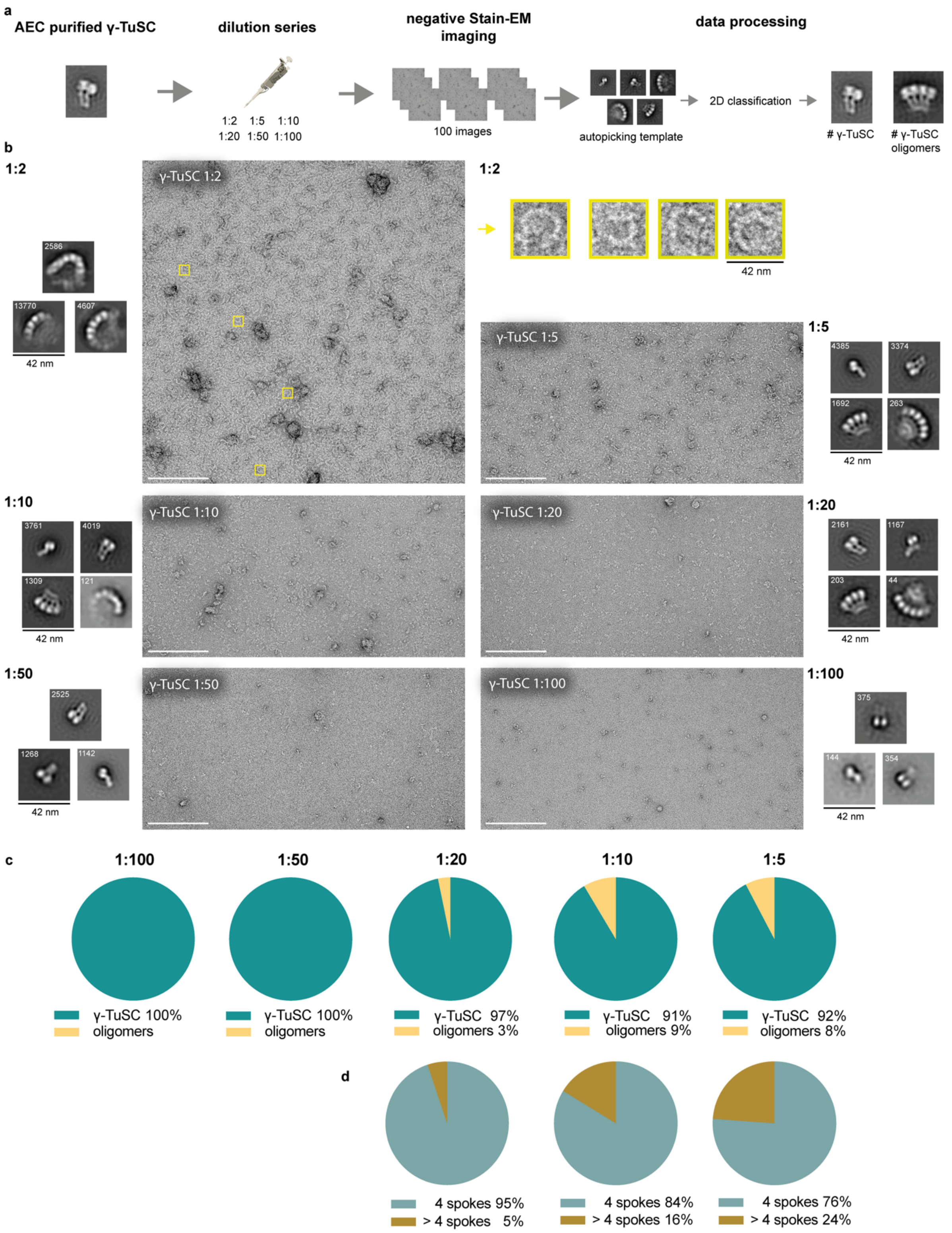

Supplementary Fig. 9: γ-TuSC oligomerization is concentration-dependent. a Experimental scheme of the γ-TuSC dilution series. AEC-purified recombinant γ-TuSC was concentrated to 3.6 µM (Amicon 30k), diluted (schematic pipette) and directly used for negative stain-EM imaging. 100 negative-stain EM images were processed in the same way for each dilution (see methods). Particles corresponding to single γ-TuSC units and γ-TuSC oligomers in the resulting 2D class averages were quantified. **b** For each dilution, a section of a representative negative stain EM micrograph is shown (scale bars 250 nm), together with representative 2D class averages of single γ-TuSC units or γ-TuSC oligomers (box sizes 42 nm). For the 1:2 dilution, zoomed individual particles are

highlighted (yellow boxes, 42 nm) additionally. Particle numbers contributing to the respective classes are given. The negative stain EM data are form one experiment, no repetition. **c,d** relative abundance of single γ-TuSC (green) units vs. γ-TuSC oligomers (yellow) (**c**), and of 4-spoked (turquoise) vs. even larger γ-TuSC oligomers (brown) (**d**). 1:100, γ-TuSC (n=873). 1:50, γ-TuSC (n=11,296). 1:20, γ-TuSC (n=25,685), oligomers (n=856). 1:10, γ-TuSC (n=30,008), oligomers (n=2,815). 1:5, γ-TuSC (n=42,055), oligomers (n=3,513). At low γ-TuSC concentration (30 nM - 60 nM, corresponding to dilution of 1:100 - 1:50), we observed exclusively individual γ-TuSC units, indicating that no oligomerization occurred. For medium γ-TuSC concentration (200 nM - 700 nM, corresponding to dilution of 1:5 - 1:20), γ-TuSC oligomers start to occur at a ratio of up to ~10% of γ-TuSC particles. **d** 1:20, 4 spokes (n=812), > 4 spokes (n=44). 1:10, 4 spokes (n=2,358), > 4 spokes (n=457). 1:5, 4 spokes (n=2,675), > 4 spokes (n=838). The maximum size of γ-TuSC oligomers increases from γ-TuSC dimers (4 spokes) up to γ-TuSC tetramers (8 spokes). At very high concentration of γ-TuSC units (1.6 µM, corresponding to dilution of 1:2), particle density was too high for comprehensive particle localization and 2D class averaging, but inspection of the micrographs suggested that a large fraction of γ-TuSC units was present in the context of very large oligomers. Source data are provided as a Source Data file.

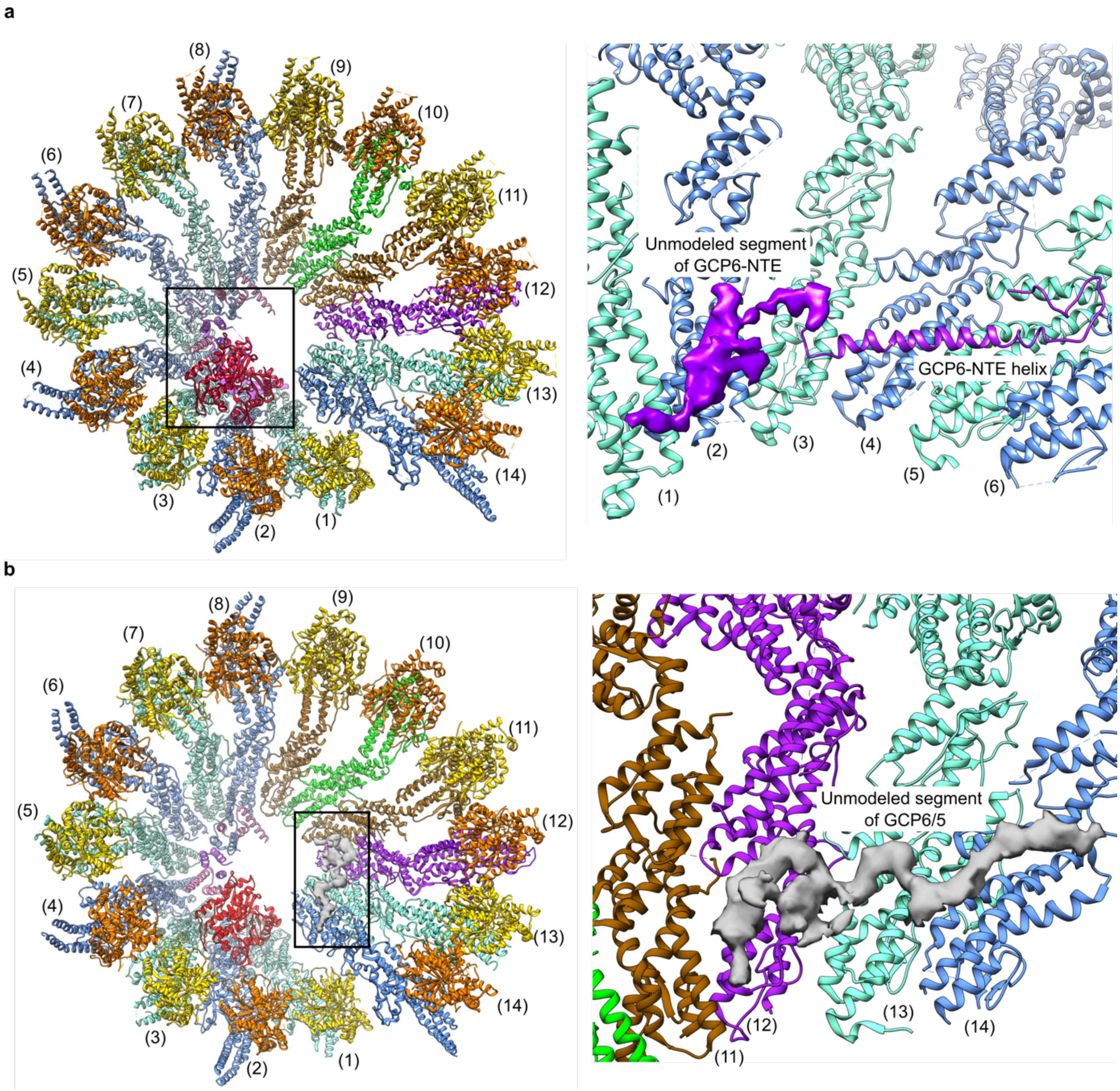

Supplementary Fig. 10: The GCP6 GRIP1-NTE interacts with most γ-TuSC units. a The GCP6 GRIP1-NTE (purple), as represented by a long helix (PBD 6X0U) and the continuous unmodeled density segment (EMD-21074), bridges and stabilizes all γ-TuSC units on the $GCP4_{(9)}$-facing side of the complex. Coloring as in Fig. 1a. **b** An unidentified density segment (grey, EMD-11888) likely corresponding either to unmodeled parts of GCP5 or GCP6 binds to and stabilizes the γ-$TuSC_{(13\text{-}14)}$ unit. Coloring as in Fig. 1a.

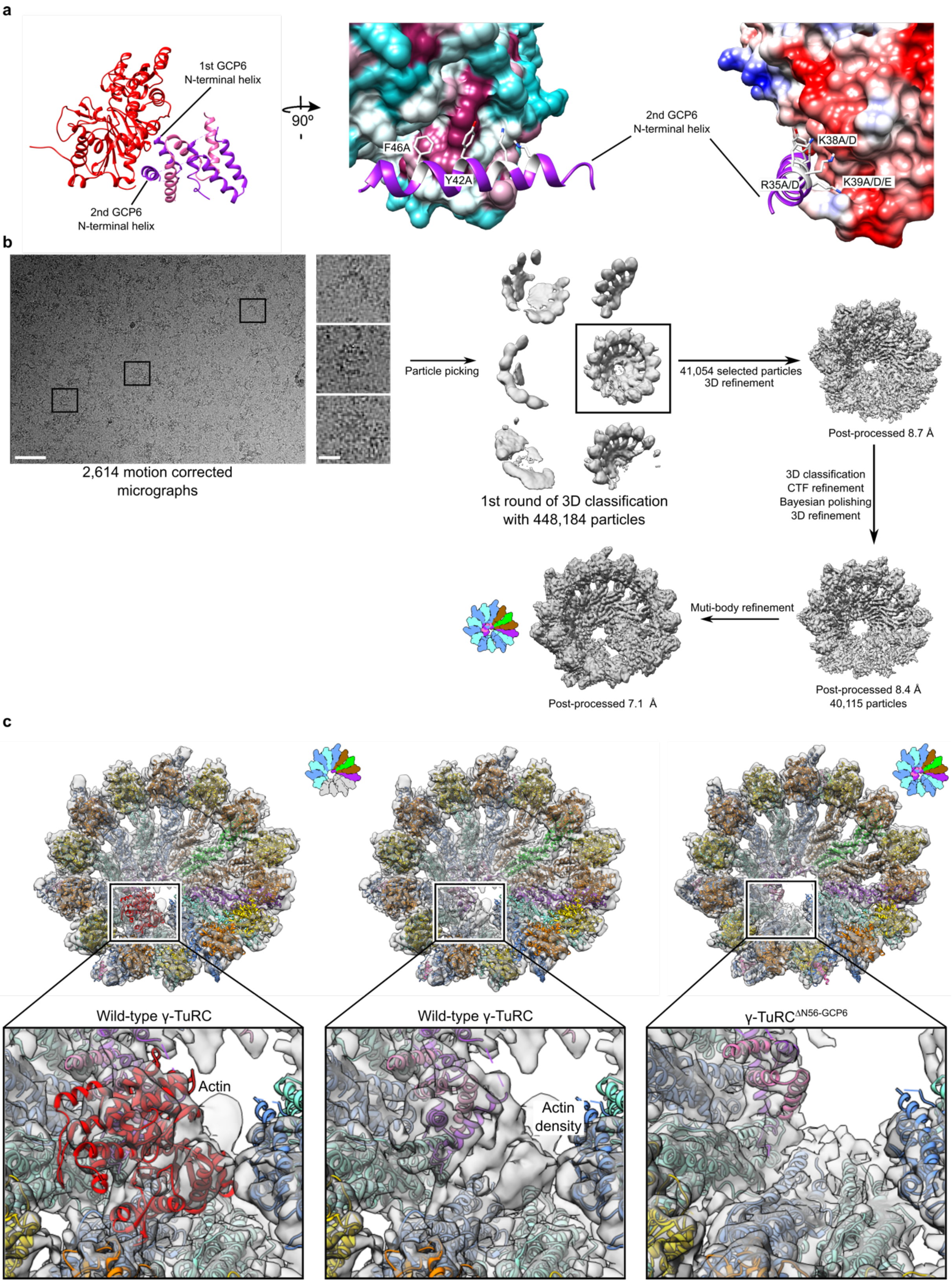

Supplementary Fig. 11: Cryo-EM processing scheme and cryo-EM reconstruction of γ-TuRC$^{\Delta N56\text{-}GCP6}$. a Left: Overview of the interface between the GCP6 GRIP1-NTE and actin. Interacting helices of the GCP6 GRIP1-NTE are indicated. Middle: Actin surface colored according to hydrophobicity (cyan: hydrophilic, white: neutral, maroon: hydrophobic). Residues of the second N-terminal GCP6 helix interacting with actin are shown in full atomic representation and colored white. Mutations of two hydrophobic residues are indicated. Right: Actin surface colored according to coulombic potential (blue: positive charge, white: neutral, red: negative charge). Mutations of three

positively charged residues are indicated. **b** Cryo-EM data processing scheme of γ-TuRC$^{ΔN56\text{-}GCP6}$. Zoom on a representative micrograph and three representative particles. Scale bars correspond to 80 nm (micrograph) and 20 nm (particles), respectively. Two consecutive rounds of 3D classification separated fully assembled γ-TuRC$^{ΔN56\text{-}GCP6}$ from false positive particles and γ-TuRC$^{ΔN56\text{-}GCP6}$ subcomplexes. Number of particles and selected classes are indicated. Selected particles from the second round of 3D classification were subjected to CTF refinement, Bayesian polishing, 3D autorefinement and multi-body refinement. Global resolution of the 3D reconstruction and particle number are indicated. **c** Comparison of cryo-EM densities obtained for recombinant wild-type γ-TuRC and recombinant γ-TuRC$^{ΔN56\text{-}GCP6}$. In the zoomed views, density segments corresponding to the lumenal bridge of the two reconstructions are compared. Data shown in **b** are from one acquisition with no repetition.

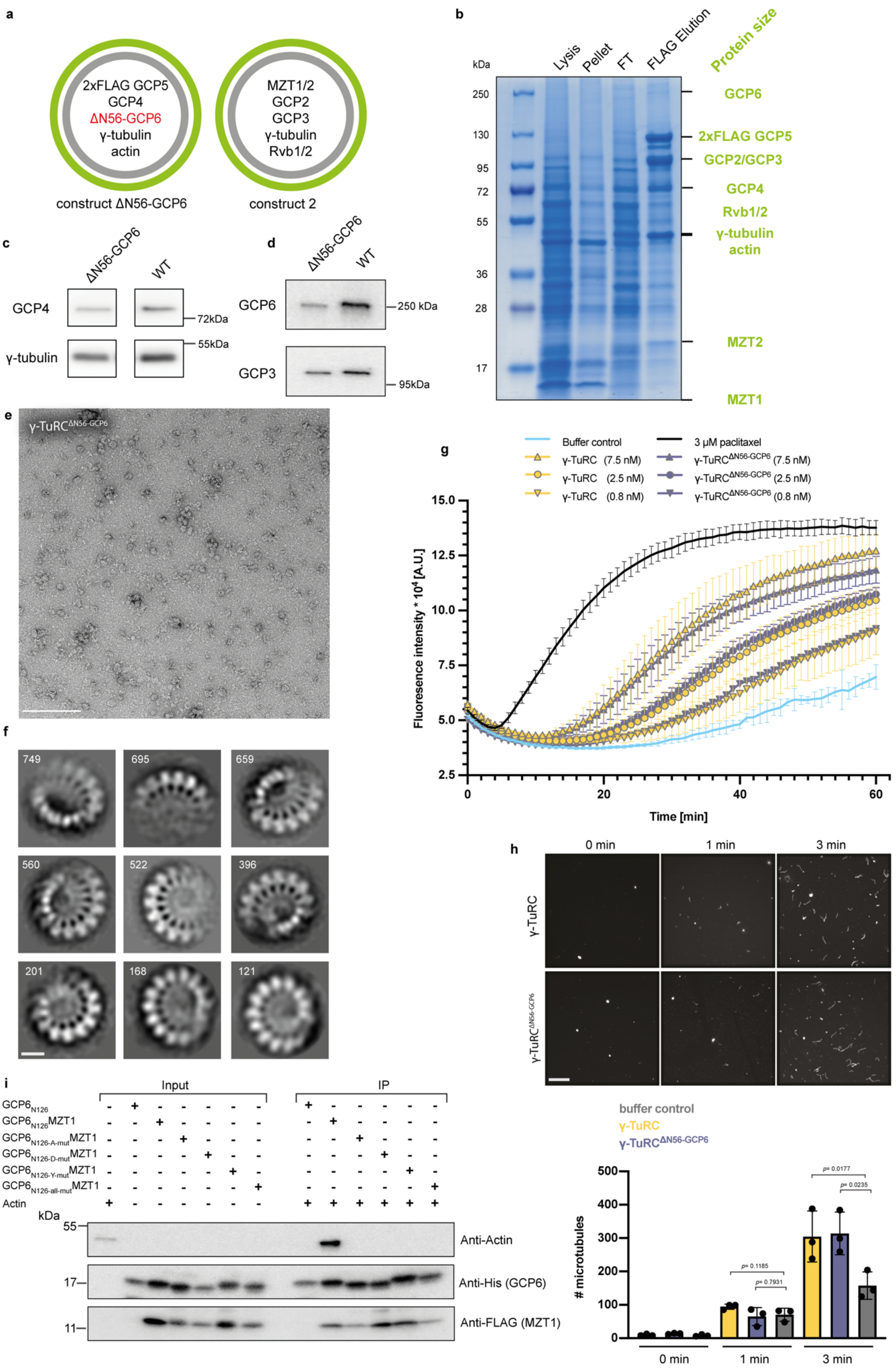
a
2xFLAG GCP5
GCP4
ΔN56-GCP6
γ-tubulin
actin
construct ΔN56-GCP6
MZT1/2
GCP2
GCP3
γ-tubulin
Rvb1/2
construct 2
b
kDa
Lysis
Pellet
FT
FLAG Elution
Protein size
GCP6
2xFLAG GCP5
GCP2/GCP3
GCP4
Rvb1/2
γ-tubulin
actin
MZT2
MZT1
c
ΔN56-GCP6
WT
GCP4
γ-tubulin
72kDa
55kDa
d
GCP6
GCP3
250 kDa
95kDa
e
γ-TuRC^ΔN56-GCP6
f
g
Buffer control
3 μM paclitaxel
Fluoresence intensity * 10^4 [A.U.]
Time [min]
h
0 min
1 min
3 min
γ-TuRC
γ-TuRC^ΔN56-GCP6
i
Input
IP
Actin
Anti-Actin
Anti-His (GCP6)
Anti-FLAG (MZT1)
buffer control
γ-TuRC
γ-TuRC^ΔN56-GCP6
microtubules

Supplementary Fig. 12: Recombinant human γ-TuRC$^{\Delta N56\text{-}GCP6}$. A Cloned MultiBac constructs (construct ΔN56-GCP6 and construct 2) with the genes of human γ-TuRC with N-terminal deletion in GCP6 (ΔN56 (red), see methods). Protein expression, purification and cryo-EM experiments were performed as described for wild-type (WT) γ-TuRC samples (Supplementary Fig. 1b). **b** Coomassie-stained SDS-PAGE of γ-TuRC$^{\Delta N56\text{-}GCP6}$ purification used for cryo-EM showing the cell lysate (lysis), cell pellet (Pellet), flowthrough (FT), and several proteins copurified with 2xFLAG GCP5 in the FLAG elution. Expected protein sizes are indicated (green). **C,d** Section of immunoblot analysis of FLAG elution of WT γ-TuRC and γ-TuRC$^{\Delta N56\text{-}GCP6}$, against GCP4/γ-tubulin (**c**), and GCP6/GCP3 (**d**) antibodies, respectively. **e** Representative negative stain EM micrograph of recombinant human γ-TuRC$^{\Delta N56\text{-}GCP6}$, scale bar 250 nm. **f** Representative negative stain EM 2D class averages of the γ-TuRC$^{\Delta N56\text{-}GCP6}$ sample. Particle numbers are given in the left upper corner of the images. Scale bar: 10 nm. **g** Batch *in vitro* microtubule nucleation assay comparing recombinant WT γ-TuRC and γ-TuRC$^{\Delta N56\text{-}GCP6}$. Shown are error bars for the standard deviation of the mean of (n=4) replicates for buffer control (blue), 3 μM Paclitaxel as positive control (black), and three different concentrations of γ-TuRC (yellow) and γ-TuRC$^{\Delta N56\text{-}GCP6}$ (purple) samples. Concentrations given in the figure correspond to the γ-tubulin concentration of the sample determined by immunoblot analysis in comparison to recombinant human γ-tubulin. **h** *In vitro* microtubule nucleation assay based on the quantification of individual microtubules. Coloring as indicated. Microtubules were nucleated either with recombinant WT γ-TuRC or γ-TuRC$^{\Delta N56\text{-}GCP6}$ at 7 nM γ-tubulin concentration (see Supplementary Fig.12g and methods), or buffer control at a αβ-tubulin concentration of 20 μM containing 5% Cy3-labelled tubulin for visualization. Upper panel: representative light microscopy images of the three timepoints (0 min, 1 min and 3 min) of the recombinant WT γ-TuRC and γ-TuRC$^{\Delta N56\text{-}GCP6}$ samples, scale bar 20 μm. Lower panel: quantification of the microtubule numbers as mean±s.d (bars) and the individual counts (dots) for each replicate of a given condition (n=3 replicates). Coloring as indicated and statistics were derived from two-tail unpaired t-test. **i** Immunoblot analysis of the actin IP experiment against His-tagged N-terminal fragments of GCP6 (GCP6$_{N126}$-His$_8$), co-expressed with *Flag-MZT1* in *E.coli*. Samples were GCP6$_{N126}$ without co-expression of *Flag-MZT1*, GCP6$_{N126}$MZT1 and constructs with point mutations predicted to interfere with actin interaction: GCP6$_{N126\text{-}A\text{-}mut}$MZT1 (R35A, K38A, K39A); GCP6$_{N126\text{-}D\text{-}mut}$MZT1 (R35D, K38D, K39D); GCP6$_{N126\text{-}Y\text{-}mut}$MZT1 (Y42A, F46A); GCP6$_{N126\text{-}all\text{-}mut}$MZT1 (R35D, K38D, K39D, Y42A, F46A). Inputs and IP samples were probed against anti-actin, anti-His (GCP6) and anti-FLAG (MZT1) antibodies. In contrast to GCP6$_{N126}$MZT1, mutated constructs do not pull down actin. Shown are sections of representative immunoblots of one experiment**,** IP experiments were performed in (n=3) independent experiments. Data shown in **b**-**f** are from one experiment with no repetition of SDS-page and negative stain EM imaging and one repetition of immunoblot analysis. Protein complex expression and purification were repeated more than n=3 times. Source data are provided as a Source Data file.

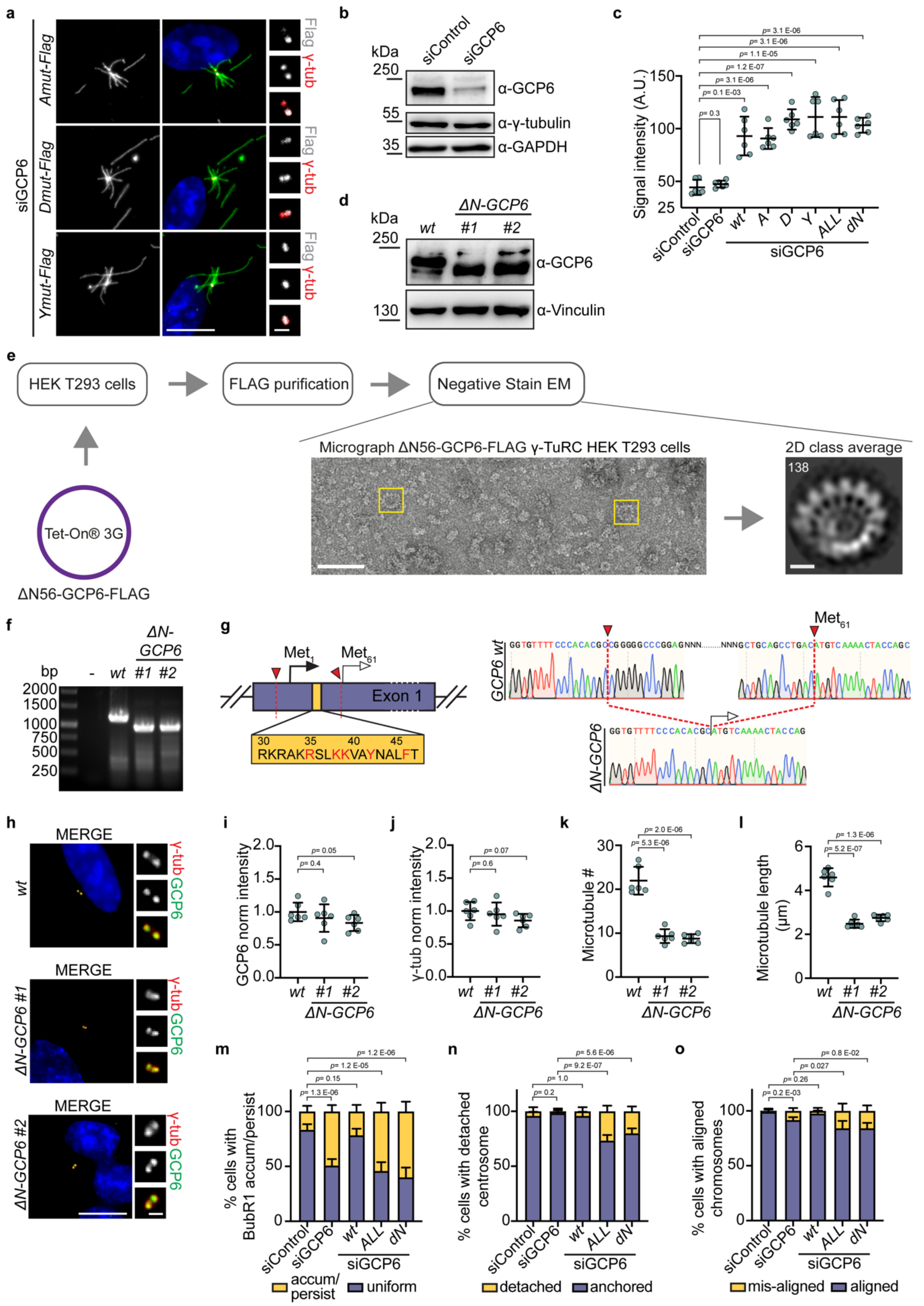

Supplementary Fig. 13: *ΔN-GCP6* cells show MT nucleation defects and mitotic progression similarly to mutant constructs. a IF of MT asters nucleated from the centrosome (γ-tubulin) in cells treated with siRNA and in which *TUBGCP6-C-terminal-FLAG* mutant constructs (*Amut* (R35A, K38A, K39A), *Dmut* (R35D, K38D, K39E) and *Ymut* (Y42A, F46A) were expressed (Flag), coloring as indicated (see Fig. 5a). **b** Immunoblot analysis of siGCP6

depletion efficiency. GCP6 depletion does not affect γ-tubulin levels in RPE1 cells. **c** FLAG signal intensity at the centrosome in the cells expressing *TUBGCP6-C-terminal-FLAG* wild-type (WT) and mutant constructs (*A* (R35A, K38A, K39A); *D* (R35D, K38D, K39E); *Y* (Y42A, F46A); *ALL* (R35D, K38D, K39E, Y42A, F46A); *dN* (ΔN56-GCP6)) quantified in IF samples of (**a**; see Fig. 5a). **d** Section of immunoblot of the endogenous GCP6 levels in *WT* and *ΔN-GCP6* (deletion of first 60 AA). Note the downshift of the GCP6 bands in the samples of the two *ΔN-GCP6* clones. **e** Negative stain EM analysis of ΔN56-GCP6-FLAG γ-TuRC purified from HEK T293 cells. Individual particles (yellow boxes) on micrographs (scale bar 100 nm) and a 2D class average (scale bar 10 nm) demonstrate that γ-TuRCs are structurally intact. **f** Genomic PCR of the *TUBGCP6* exon 1 locus targeted by the sgRNAs to generate the *ΔN-GCP6* clones. The genomic PCR product in the *ΔN-GCP6* clones reflect the calculated loss of 205 nt. **g** Left: scheme of the *ΔN-GCP6* cell line generation. The red arrowheads point the sgRNA target sites in Exon 1 (purple) with the actin interacting region (yellow). The black arrow represents the endogenous start codon Met_1, while the white one the resulting start codon (Met_{61}) in *ΔN-GCP6*. Right: the sequenced genomic region of the WT and *ΔN-GCP6* cells, coloring as indicated. **h-j** *ΔN-GCP6* clones do not show a significant decrease of both γ-tubulin and GCP6 endogenous levels. **i** Quantification of the GCP6 signal intensity in IF (**h**). **j** Quantification of the γ-tubulin signal intensity in IF (**h**). Coloring as indicated. **k,l** *In vivo* MT regrowth assay in *WT* and *ΔN-GCP6* RPE1 cells. Average number (**k**) and average size (**l**) of microtubules nucleated from the centrosome in *WT* and *ΔN-GCP6* RPE1 cells. **m** Quantification of metaphase cells (%) in which BubR1 accumulated/persisted on centromeres non-uniformly in cells treated with siGCP6 and in which *WT* and *TUBGCP6* mutant constructs were expressed. **n** Quantification of metaphase cells (%) in which centrosomes detached from the spindle pole in cells treated with siGCP6 and in which *WT* and *TUBGCP6* mutant constructs were expressed. **o** Quantification of metaphase cells (%) in which chromosomes were aligned or close to alignment in the metaphase plate in cells treated with siGCP6 and in which *WT* and *TUBGCP6* mutant constructs were expressed. **m-o** Coloring as indicated and naming of mutants as in (**c**), (**a** and **h** scale bars: 10 µm; magnification scale bar: 1 µm; **c**, **i-o** data are presented as mean±s.d., all statistics were derived from two-tail unpaired t-test analysis of: 3 replicates of 2 independent experiment (n=6)). Immunoblots shown in **b**,**c** and agarose gel in **f** are representative of n=2 experiments, negative stain EM data shown in **e** are from one experiment with no repetition. Source data are provided as a Source Data file.

Supplementary Table 1: Cryo-EM data collection and refinement statistics

Data collection and processing			
Sample	γ-TuRC WT	γ-TuRC$^{\Delta N56\text{-}GCP6}$	γ-TuRC$^{\Delta GCP2/3}$
EMDB ID	14005, 14006, 14007, 14008, 14009, 14010, 14011, 14012, 14013, 14014, 14015, 14016, 14017	14018	14019
PDB ID	7QJ0, 7QJ1, 7QJ2, 7QJ3, 7QJ4, 7QJ5, 7QJ6, 7QJ7, 7QJ8, 7QJ9, 7QJA, 7QJB, 7QJC	7QJD	7QJE
Microscope	Titan Krios G1	Titan Krios G1	Titan Krios G1
Detector	Gatan K3 camera	Gatan K3 camera	Gatan K3 camera
Magnification	33,000	33,000	81,000
Voltage (kV)	300	300	300
Electron exposure (e^-/Å^2)	35	42.6	62.4
Defocus range (μm)	-2 to -2.5	-2 to -2.5	-1 to -3
Pixel size (Å/px)	2.66	2.66	1.07
Symmetry imposed	C1	C1	C1
Number of movies	6,127	2,614	11,276
Initial particle images	436,887	448,184	1,888,166
Final particle images	92,149; 59,861; 10,965; 17,337; 6,097; 36,881; 28,611; 8,270; 5,443; 21,860; 5,376; 6,456; 2,645	40,115	9,192
Map resolution (Å)	5.3, 7.0, 8.6, 7.6, 9.0, 8.7, 7.8, 8.7, 8.7, 8.1, 9.2, 9.0, 16.1	7.1	7.8
FSC threshold	0.143	0.143	0.143
Map-sharpening B factor (Å^2)	-248.2, -475.1, -410.5, -366.7, -544.1, -434.4, -378.7, -249.2, -201.6, -553.1, -300.0, -300.0, -45.47	-282.3	-687.1
Model building and refinement			
Model composition			
Protein atoms	51,018; 50,991; 69,211; 69,169; 87,398; 126,797; 91,728; 109,757; 108,530; 91,688; 109,749; 108,550; 125,300	124,856	31,375
Nucleic acid atoms	0, 0, 0, 0, 0, 0, 0, 0, 0, 0, 0, 0, 0	0	0
Ligands	0, 0, 0, 0, 0, 0, 0, 0, 0, 0, 0, 0, 0	0	0
RMSDs from ideal			
Bond lengths (Å)	0.007, 0.006, 0.007, 0.007, 0.008, 0.007, 0.007, 0.006, 0.006, 0.007, 0.006, 0.006, 0.006	0.007	0.006
Bond angles (°)	1.149, 1.310, 1.271, 1.370, 1.269, 1.318, 1.229, 1.163, 1.152, 1.290, 1.171, 1.170, 1.179	1.177	1.276
Validation			
Clashscore	4.59, 4.88, 5.29, 5.96, 6.44, 6.60, 5.52, 5.71, 4.97, 6.65, 4.41, 4.81, 6.26	5.51	5.00

Rotamer outliers (%)	4.71, 8.34, 6.37, 10.49, 6.35, 6.21, 5.38, 4.65, 3.42, 5.62, 4.12, 3.75, 4.47	3.15	0.55
Ramachandran plot			
Favored (%)	95.25, 95.79, 95.30, 95.70, 95.01, 94.61, 94.23, 94.95, 94.46, 94.79, 94.90, 94.58, 95.07	95.34	92.91
Allowed (%)	4.44, 3.95, 4.52, 3.91, 4.50, 5.08, 5.30, 4.81, 5.11, 4.94, 4.86, 5.26, 4.54	4.41	6.23
Outlier (%)	0.31, 0.26, 0.18, 0.39, 0.49, 0.31, 0.47, 0.24, 0.43, 0.26, 0.23, 0.16, 0.39	0.25	0.86

Supplementary Table 2: Primer list.

Name	Sequence (5' → 3')	Source	Identifier
MZT2_fwd	AAAACCTATAAATATATGGCGGCGCAGGGCGTA	This study	N/A
MZT2_rev	ACTGCAGGCTCTAGACTAGGTGCTGCCCCGTGTAG	This study	N/A
GCP3_fwd	AAAACCTATAAATATGGCGACCCCGGACCAGAAG	Würtz et al., 2021[3]	N/A
GCP3-TEV-2xFLAG_rev	GATTACTTATCGTCGTCATCCTTGTAATCCTTGTCG TCATCGTCCTTGTAGTCTCCGCCTCCTCCGCCGCC CTGAAAATACAGGTTTTCGGATGCCGTGTGGGAGC TGCGCCGCC	This study	N/A
ΔN56-GCP6_fwd	CATATTTATAGGTTTTTTATTACAAAACTGTTACGA	This study	N/A
ΔN56-GCP6_rev	TCTAGAGCCTGCAGTCTCG	This study	N/A
MultiBac_vector_fwd	TCTAGAGCCTGCAGTCTCG	Würtz et al., 2021	N/A
MultiBac_vector_rev	CAGTTTTGTAATAAAAAACCTATAAATAT	Würtz et al., 2021	N/A
Combination_vector_fwd	TTCGCGACCTACTCCGGA	Würtz et al., 2021	N/A
Combination_vector_rev	CAGATAACTTCGTATAATGTATGCT	Würtz et al., 2021	N/A
Combination_insert_fwd	ATACGAAGTTATCTGTTCGCGACCTACTCCGGA	Würtz et al., 2021	N/A
Combination_insert_rev	GGAGTAGGTCGCGAAGATCCAGACATGATAAGATA CATTG	Würtz et al., 2021	N/A
FLAG-MZT1 fwd	ACTTTAAGAAGGAGATATACCATGGATGGACTACAA AGACGATGACGACAAGGGTTCTATGGCGAGTAGCG GCGGT	This study	N/A
FLAG-MZT1 rev	CTGCAGGCGCGCCGAGCTCGAATTCTCAGCTTGTC ATATTTTCAGCAGCCTTC	This study	N/A
GCP6N126 fwd	GTATAAGAAGGAGATATACATATGGCCAGCATCAC GCAGCTG	This study	N/A
GCP6N126 rev	CAGCGGTTTCTTTACCAGACTCGAGTTAATGATGAT GGTGGTGGTGATGATGGCCGGACAGAACTTGAGG GGGACCAC	This study	N/A
GCP6(pET26b)_fwd	CTTTAAGAAGGAGATATACATATGGCCAGCATCAC GCAGCTG	This study	N/A
GCP6(pET26b)_rev	GGTGATGGTGATGGTGGTGGGATCCCAGAACTTGA GGGGGACCAC	This study	N/A
42A 46A fwd	GTGGCCGCTAATGCTCTTGCGACAAATCTTTTTCAA GATGAGACTCAAC	This study	N/A
42A 46A rev	GCATTAGCGGCCACCTTCTTGAGGCT	This study	N/A
35D 38D 39D fwd	AGGGCAAAGGATAGCCTCGACGACGTGGCCTACA ATGCTCTTTTC	This study	N/A
35D 38D 39D rev	AGGCTATCCTTTGCCCTCTTCCGGTT	This study	N/A
35D 38D 39D for all mut	AGGGCAAAGGATAGCCTCGACGACGTGGCCGCTA ATGCTCTTGCGACA	This study	N/A

35A 38A 39A for all fwd	GCAAAGGCGAGCCTCGCTGCCGTGGCCGCTAATGCT	This study	N/A
35A 38A 39A for all rev	AGCATTAGCGGCCACGGCAGCGAGGCTCGCCTTTGC	This study	N/A
35A 38A 39A fwd	GAAGAGGGCAAAGGCGAGCCTCGCGGCGGTGGCCTACAATGCTCTTTTC	This study	N/A
35A 38A 39A rev	AGGCTCGCCTTTGCCCTCTTCCGGTT	This study	N/A
ΔN56-GCP6 (pETDUET) fwd	GAAGGAGATATACATATGCTGCAGCCTGACATGTCAAA	This study	N/A
ΔN56-GCP6 (pETDUET) rev	ATGTATATCTCCTTCTTATACTTAACTAATATACTAAGATGG	This study	N/A
pRetroX-TRE3G-hGCP6siRES-linkerFlag fw	CAACACTTTTGTCTTATACTTGGATCCATCGATAGCCACCATGGCCAGCATCACGC	This study	N/A
pRetroX-TRE3G-hGCP6-linkerFlag siRES mut rv	GTAATTGCTGGGTTTCGTCTTGAAAAAGATTTGTGAAAAGAGCATTGTAGG	This study	N/A
pRetroX-TRE3G-hGCP6-linkerFlag siRES mut fw	CAAATCTTTTTCAAGAcGAaACcCAgCAatTaCAGCCTGACATGTCAAAACTACC	This study	N/A
pRetroX-TRE3G-hGCP6siRES-linkerFlag rv	CTGCACCTGCACCAGCTCCTGCGGCGTCCTGGTAGTAGTTG	This study	N/A
pRetroX-TRE3G-hGCP6-linkerFlag R35D K38D K39E mut rv	CTCGTCGAGGCTGTCCTTTGCCCTCTTCCGGTTC	This study	N/A
pRetroX-TRE3G-hGCP6-linkerFlag R35D K38D K39E mut fw	GGACAGCCTCGACGAGGTGGCCTACAATGCTC	This study	N/A
pRetroX-TRE3G-hGCP6-linkerFlag R35A K38A K39A mut rv	AGCAGCGAGGCTGGCCTTTGCCCTCTTCCGGTTC	This study	N/A
pRetroX-TRE3G-hGCP6-linkerFlag R35A K38A K39A mut fw	GCCAGCCTCGCTGCTGTGGCCTACAATGCTCTTTTCAC	This study	N/A
pRetroX-TRE3G-hGCP6-linkerFlag Y42A F46A mut rv	TGGCAAGAGCATTGGCGGCCACCTTCTTGAGGCTC	This study	N/A
pRetroX-TRE3G-hGCP6-linkerFlag Y42A F46A mut fw	CGCCAATGCTCTTGCCACAAATCTTTTTCAAGACGAAACCCAGCAATTAC	This study	N/A

pRetroX-TRE3G-hGCP6-linkerFlag R35D K38D K39E Y42A F46A mut rv	TGGCAAGAGCATTGGCGGCCACCTCGTCGAGG	This study	N/A
pRetroX-TRE3G-hGCP6-linkerFlag R35D K38D K39E Y42A F46A mut fw	CGCCAATGCTCTTGCCACAAATCTTTTTCAAGACGAAACCCAGC	This study	N/A
pRetroX-TRE3G-hGCP6siRES-linkerFlag deltaN fw	CAACACTTTTGTCTTATACTTGGATCCATCGATAGCCACCATGtTaCAGCCTGACATGTCAAAACTAC	This study	N/A
sgRNA hGCP6 cut1 fw	caccgGGTGTTTTCCCACACGCCGG	This study	N/A
sgRNA hGCP6 cut1 rv	aaacCCGGCGTGTGGGAAAACACCc	This study	N/A
sgRNA hGCP6 cut2 fw	caccgGCTGGTAGTTTTGACATGTC	This study	N/A
sgRNA hGCP6 cut2 rv	aaacGACATGTCAAAACTACCAGCc	This study	N/A
hGCP6 KO PCR fw	GTGGGCACTTTCCACGGGTGAG	This study	N/A
hGCP6 KO PCR rv	GAAGAGCGAGACCCGGGTGTCT	This study	N/A

Supplementary Table 3: Antibodies.

Antibody	Source	Identifier
Rabbit anti-GAPDH	CellSignalling	14C10
Mouse anti-Vinculin	Proteintech	66305-1-Ig
Mouse monoclonal anti-β-Actin (human)	Proteintech	66009-1-Ig
Mouse anti-γ-tubulin	Abcam	Ab27074
Guinea pig anti-γ-tubulin	Atorino et al. 2020[4]	N/A
Rabbit anti-TUBGCP3	Proteintech	15719-1-AP
Rabbit anti-FL-GCP4	Liu et al. 2019[5]	N/A
Rabbit polyclonal anti-GCP6	Bethyl	A302-662A
Mouse monoclonal HRP conjugated anti-penta-His	Proteintech	HRP-66005
Mouse anti-penta-His	QIAGEN	34660
Rabbit Polyclonal DDDDK tag (FLAG)	Proteintech	20543-1-AP
Mouse anti-FLAG	Cell signaling	9A3
Rabbit anti-α-tubulin	MBL	PM054
Mouse anti-α-tubulin	SigmaAldrich	DM1A
Mouse anti-BubR1	Abcam	Ab4637
Rabbit anti-Pericentrin	Abcam	Ab4448

References

1. Thomas, W. & Kelley, C. Gnuplot 5.2: an interactive plotting program. (2019).
2. Wieczorek, M. *et al.* Asymmetric Molecular Architecture of the Human γ-Tubulin Ring Complex. *Cell* **180**, 165-175.e16 (2020).
3. Würtz, M. *et al.* Reconstitution of the recombinant human γ-tubulin ring complex. *Open Biol.* **11**, 200325 (2021).
4. Atorino, E. S., Hata, S., Funaya, C., Neuner, A. & Schiebel, E. CEP44 ensures the formation of bona fide centriole wall, a requirement for the centriole-to-centrosome conversion. *Nat. Commun.* **11**, 903 (2020).
5. Liu, P. *et al.* Insights into the assembly and activation of the microtubule nucleator γ-TuRC. *Nature* (2019) doi:10.1038/s41586-019-1896-6.

Supplementary source data - Gels

Related to Supplementary Fig. S1c

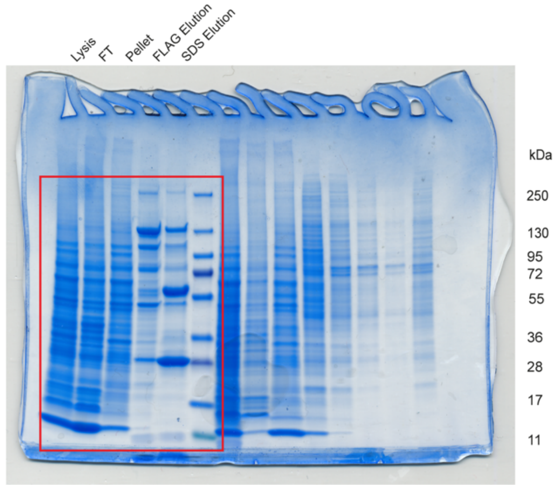

Related to Supplementary Fig. S6b

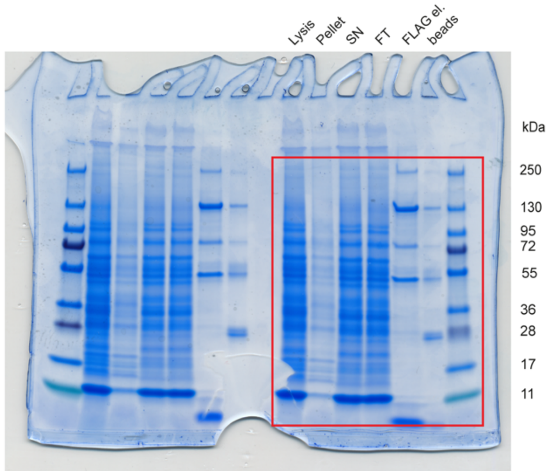

Related to Supplementary Fig. S8d

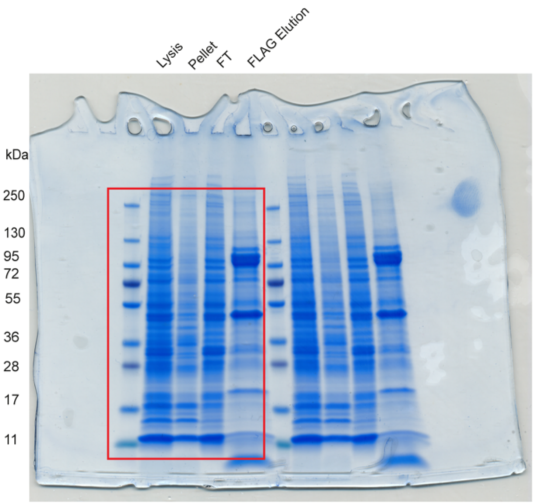

Related to Supplementary Fig. S8e

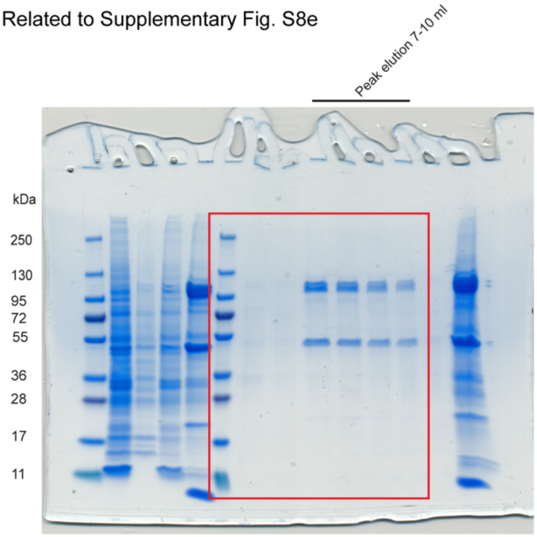

Related to Supplementary Fig. S12b

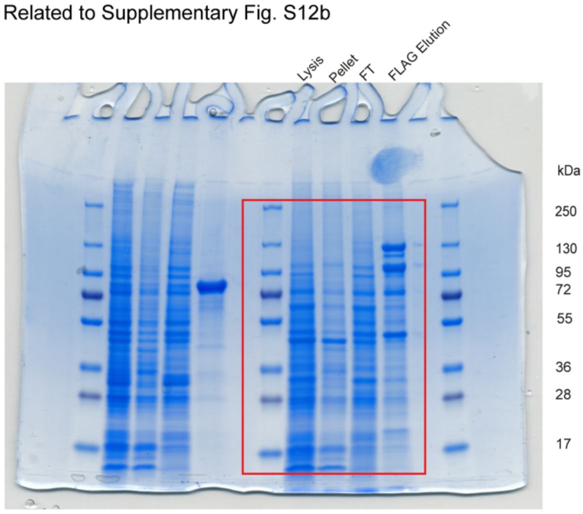

Related to Supplementary Fig. S6c

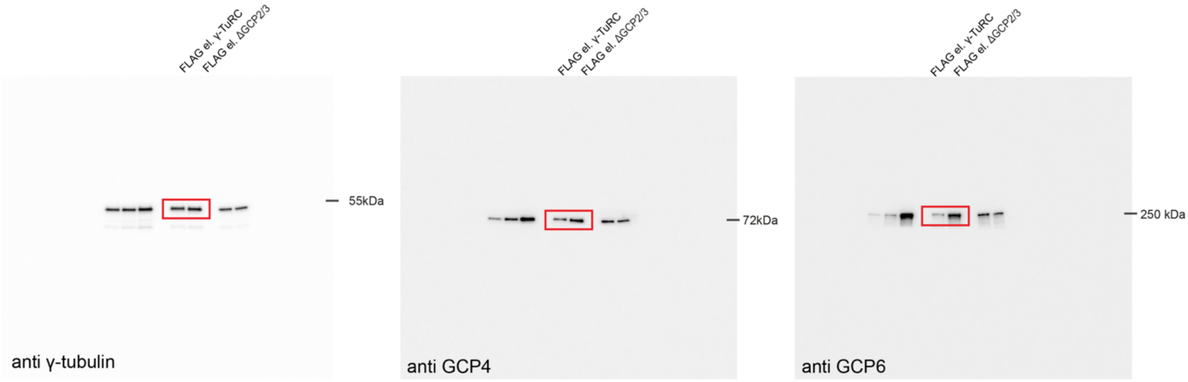

Related to Supplementary Fig. S12c

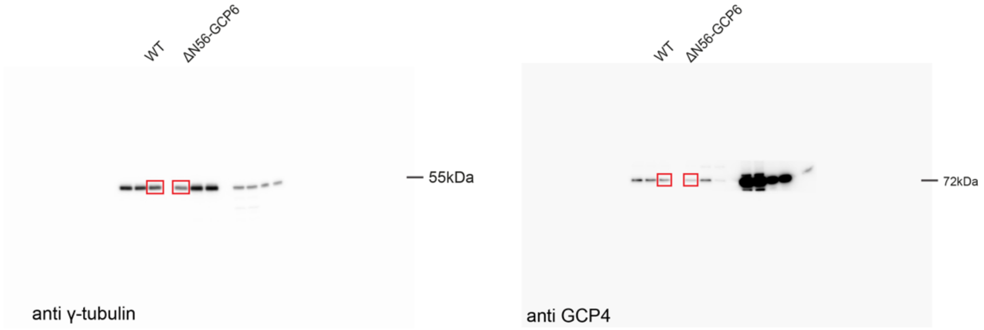

Related to Supplementary Fig. S12d

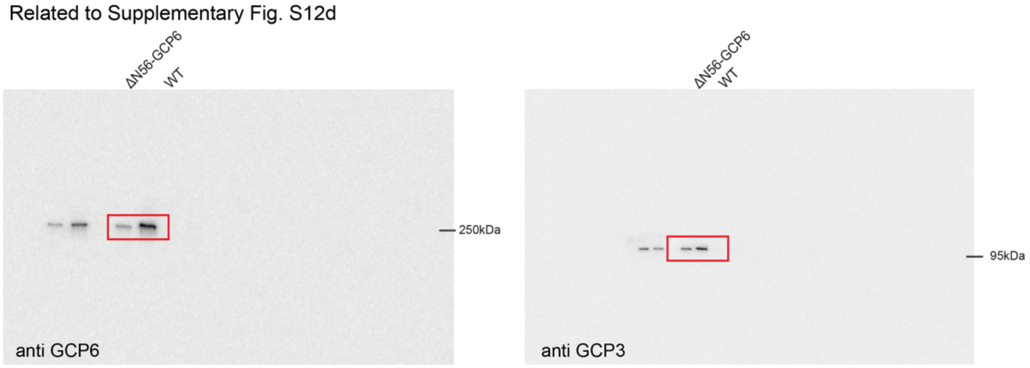

Related to Supplementary Fig. S12i

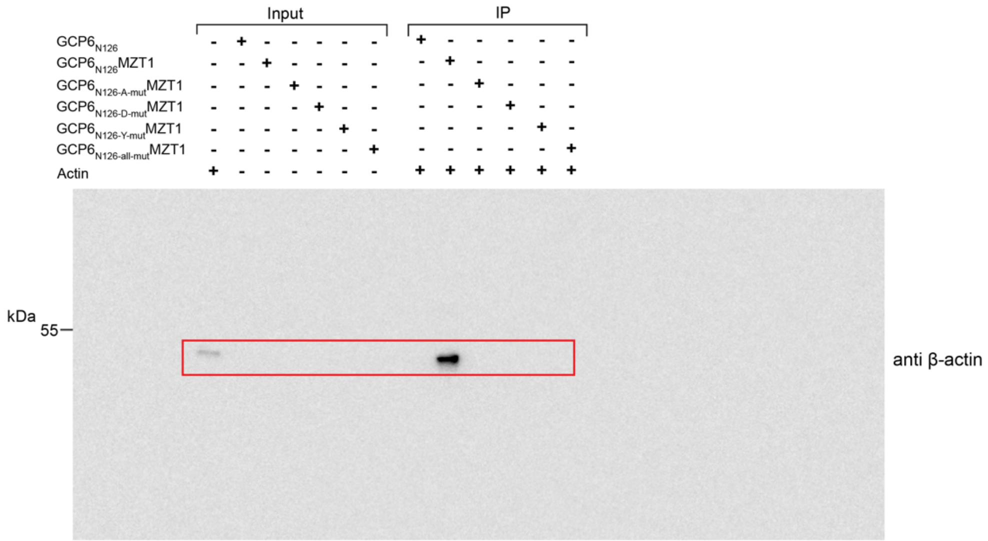

Related to Supplementary Fig. S12i

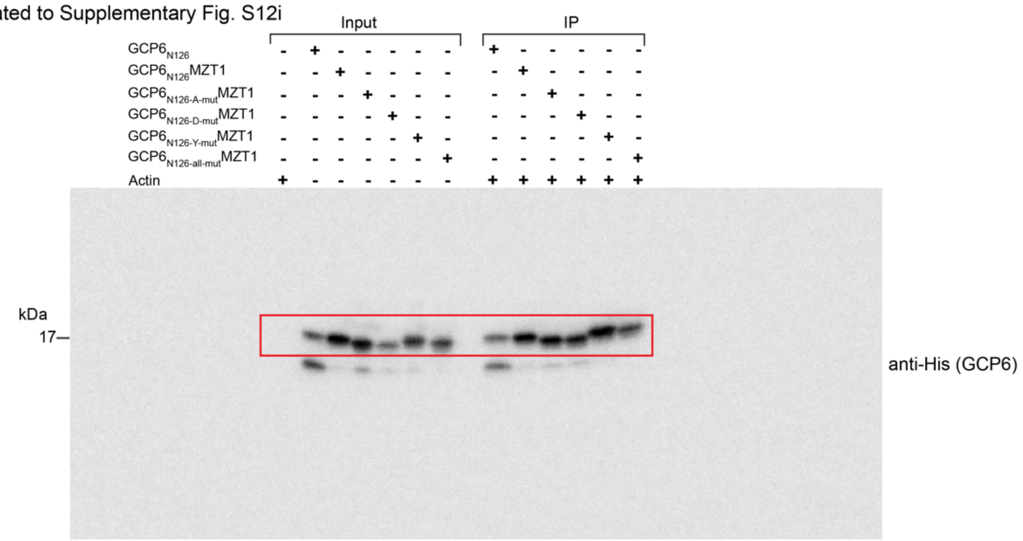

Related to Supplementary Fig. S12i

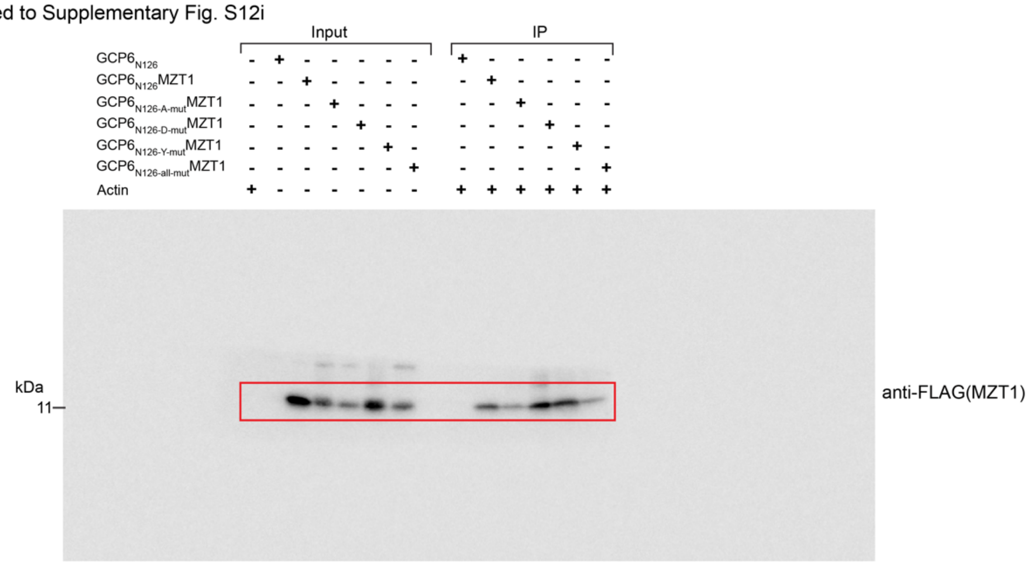

Related to Supplementary Fig. S13b

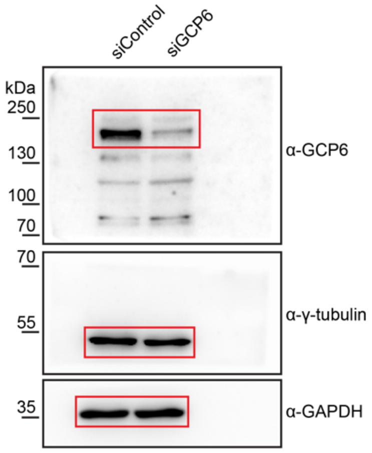

Related to Supplementary Fig. S13d

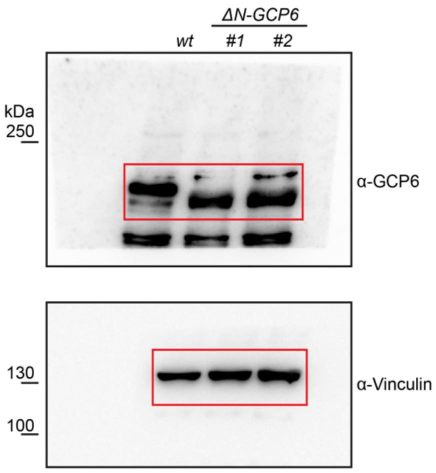

Related to Supplementary Fig. S13f

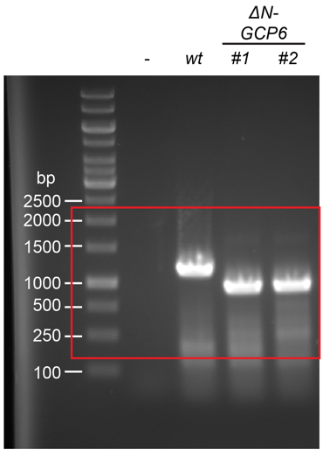

Supplement: Supplementary file 1 — Supplementary Information [file 41467_2022_28079_MOESM1_ESM.pdf]
