# Supplementary material for: Modular assembly of the principal microtubule nucleator γ-TuRC
Source: Nat Commun. 2022 Jan 25;13:473. doi: 10.1038/s41467-022-28079-0 (PMC8789826; doi:10.1038/s41467-022-28079-0)
Supplement: Supplementary file 3 — Description of Additional Supplementary Files [file 41467_2022_28079_MOESM3_ESM.pdf]

Supplementary Movie 1: Time-lapse imaging of RPE1 *TUBG1-mRuby2 (green pseudo-color) / mNeonGreen-LMNB1 (red pseudo-color)* cell undergoing mitosis. The imaged mitosis events show centrosome cohesion (green, -00:08 h:min) followed by centrosome separation (green, 00:00 h:min), nuclear envelope breakdown (red, 01:32 h:min) and nuclear envelope re-formation (red, 01:48 h:min). 4 min interval between frames.

Supplementary Movie 2: Time-lapse imaging of RPE1 ΔN-GCP6 clone #1, TUBG1-mRuby2 (green pseudo-color) / mNeonGreen-LMNB1 (red pseudo- color) cell undergoing mitosis. The imaged mitosis events show centrosome cohesion (green, -00:08 h:min) followed by centrosome separation (green, 00:00 h:min), nuclear envelope breakdown (red, 01:56 h:min) and nuclear envelope re-formation (red, 02:12 h:min). 4 min interval between frames.

Supplementary Movie 3: Time-lapse imaging of RPE1 ΔN-GCP6 clone #2, TUBG1-mRuby2 (green pseudo-color) / mNeonGreen-LMNB1 (red pseudo-color) cell undergoing mitosis. The imaged mitosis events show centrosome cohesion (green, -00:08 h:min) followed by centrosome separation (green, 00:00 h:min), nuclear envelope breakdown (red, 02:24 h:min) and nuclear envelope re-formation (red, 02:56 h:min). 4 min interval between frames.
