# Supplementary material for: Modular assembly of the principal microtubule nucleator γ-TuRC
Source: Nat Commun. 2022 Jan 25;13:473. doi: 10.1038/s41467-022-28079-0 (PMC8789826; doi:10.1038/s41467-022-28079-0)
Supplement: Supplementary file 7 — Reporting Summary [file 41467_2022_28079_MOESM7_ESM.pdf]

nature research

Corresponding author(s): Stefan Pfeffer, Elmar Schiebel

Last updated by author(s): Dec 20, 2021

Reporting Summary

Nature Research wishes to improve the reproducibility of the work that we publish. This form provides structure for consistency and transparency in reporting. For further information on Nature Research policies, see our Editorial Policies and the Editorial Policy Checklist.

Statistics

For all statistical analyses, confirm that the following items are present in the figure legend, table legend, main text, or Methods section.

n/a	Confirmed	
☐	☒	The exact sample size (*n*) for each experimental group/condition, given as a discrete number and unit of measurement
☐	☒	A statement on whether measurements were taken from distinct samples or whether the same sample was measured repeatedly
☐	☒	The statistical test(s) used AND whether they are one- or two-sided<br>*Only common tests should be described solely by name; describe more complex techniques in the Methods section.*
☒	☐	A description of all covariates tested
☒	☐	A description of any assumptions or corrections, such as tests of normality and adjustment for multiple comparisons
☐	☒	A full description of the statistical parameters including central tendency (e.g. means) or other basic estimates (e.g. regression coefficient) AND variation (e.g. standard deviation) or associated estimates of uncertainty (e.g. confidence intervals)
☐	☒	For null hypothesis testing, the test statistic (e.g. *F*, *t*, *r*) with confidence intervals, effect sizes, degrees of freedom and *P* value noted<br>*Give P values as exact values whenever suitable.*
☒	☐	For Bayesian analysis, information on the choice of priors and Markov chain Monte Carlo settings
☒	☐	For hierarchical and complex designs, identification of the appropriate level for tests and full reporting of outcomes
☒	☐	Estimates of effect sizes (e.g. Cohen's *d*, Pearson's *r*), indicating how they were calculated

Our web collection on statistics for biologists contains articles on many of the points above.

Software and code

Policy information about availability of computer code

Data collection	Datasets for cryo-EM were collected with EPU (version 2.6) and γ-TuRCΔGCP2/3 EPU (version 2.9) at Krios TEM equipped with Gatan K3 camera operated by Gatan Microscopy Suite (version 3.32). Datasets for negative stain were collected at Talos L120C equipped with Ceta 16M using Thermofischer Scientific software EPU 2.9 . Anion exchange chromatography was performed at Äkta go operated by Unicorn (version 7.5). Immuno-Fluorescence data were acquired with softWoRx v6.1.1 Release 5 (Applied Precision, GE), Immuno-blots were acquired with LAS4000IR v2.1 software, while agarose gel with Quantity One v4.6.9 (BioRad).
Data analysis	Cryo-EM data were processed using Relion 3.1, Relion 3.0-Beta, Topaz 0.2.4, CryoSPARC 3.2 , MotionCorr2 v1.0.5. and gCtf 1.06. All density map-related figures were prepared in UCSF Chimera 1.13.1 and UCSF ChimeraX 1.1.1. Atomic building of the model was performed in Coot 0.9. Refinement and flexible fitting of the model was performed using the VMD (version 1.9.3), QwikMD v1.0, MDFF (version 0.4), NAMD (version 2.14), Namdinator v2.0, Phenix 1.14 and MolProbity. Analysis and vector visualisation of γ-TuSC conformations was performed in PyMOL 2.1. Negative stain data were processed using Relion 3.1, gCtf 1.06. FSC curves were plotted in Gnuplot (version 5.2). Immuno-fluorescence, Immuno-blots, agarose gels were analyzed by Fiji (ImageJ v2.1.0/1.53c). Prism v9.2.0 (283) (GraphPad Software) was used for the data representation and mean and standard deviation values calculation. Microsoft Excel (v16.46.21021202) was used for p-values calculation and data pools normalization. Sequencing data of the ΔN-GCP6 knock out cell lines were analyzed with SnapGene v5.3.2.

For manuscripts utilizing custom algorithms or software that are central to the research but not yet described in published literature, software must be made available to editors and reviewers. We strongly encourage code deposition in a community repository (e.g. GitHub). See the Nature Research guidelines for submitting code & software for further information.

Data

Policy information about availability of data

All manuscripts must include a data availability statement. This statement should provide the following information, where applicable:

- Accession codes, unique identifiers, or web links for publicly available datasets
- A list of figures that have associated raw data
- A description of any restrictions on data availability

Atomic coordinates and the associated cryo-EM densities for assembly intermediates of the recombinant wild-type γ-TuRC have been deposited in the Protein Data Bank and the Electron Microscopy Data Bank under accession codes PDB-7QJ0 [https://www.rcsb.org/structure/7QJ0] /EMD-14005 [https://www.ebi.ac.uk/pdbe/entry/emdb/EMD-14005], PDB-7QJ1 [https://www.rcsb.org/structure/7QJ1] /EMD-14006 [https://www.ebi.ac.uk/pdbe/entry/emdb/EMD-14006], PDB-7QJ2 [https://www.rcsb.org/structure/7QJ2] /EMD-14007 [https://www.ebi.ac.uk/pdbe/entry/emdb/EMD-14007], PDB-7QJ3 [https://www.rcsb.org/structure/7QJ3] / EMD-14008 [https://www.ebi.ac.uk/pdbe/entry/emdb/EMD-14008], PDB-7QJ4 [https://www.rcsb.org/structure/7QJ4] /EMD-14009 [https://www.ebi.ac.uk/pdbe/entry/emdb/EMD-14009], PDB-7QJ5 [https://www.rcsb.org/structure/7QJ5] /EMD-14010 [https://www.ebi.ac.uk/pdbe/entry/emdb/EMD-14010], PDB-7QJ6 [https://www.rcsb.org/structure/7QJ6] /EMD-14011 [https://www.ebi.ac.uk/pdbe/entry/emdb/EMD-14011], PDB-7QJ7 [https://www.rcsb.org/structure/7QJ7] / EMD-14012 [https://www.ebi.ac.uk/pdbe/entry/emdb/EMD-14012], PDB-7QJ8 [https://www.rcsb.org/structure/7QJ8] /EMD-14013 [https://www.ebi.ac.uk/pdbe/entry/emdb/EMD-14013], PDB-7QJ9 [https://www.rcsb.org/structure/7QJ9] /EMD-14014 [https://www.ebi.ac.uk/pdbe/entry/emdb/EMD-14014], PDB-7QJA [https://www.rcsb.org/structure/7QJA] /EMD-14015 [https://www.ebi.ac.uk/pdbe/entry/emdb/EMD-14015], PDB-7QJB [https://www.rcsb.org/structure/7QJB] / EMD-14016 [https://www.ebi.ac.uk/pdbe/entry/emdb/EMD-14016], PDB-7QJC [https://www.rcsb.org/structure/7QJC] /EMD-14017 [https://www.ebi.ac.uk/pdbe/entry/emdb/EMD-14017]. Atomic coordinates and the associated cryo-EM densities for recombinant actin binding-deficient γ-TuRC and the recombinant wild-type 4-spoke assembly intermediate have been deposited in the Protein Data Bank and the Electron Microscopy Data Bank under accession codes, PDB-7QJD [https://www.rcsb.org/structure/7QJD] /EMD-14018 [https://www.ebi.ac.uk/pdbe/entry/emdb/EMD-14018], PDB-7QJE [https://www.rcsb.org/structure/7QJE] /EMD-14019 [https://www.ebi.ac.uk/pdbe/entry/emdb/EMD-14019], respectively. The raw cryo-EM micrograph movie stacks are available from the corresponding authors only upon request due to the large size of the datasets. Source data are provided with this paper. Constructs generated in this study are available upon request to the corresponding authors. Published structural data used in this article: PDB-6X0U [https://www.rcsb.org/structure/6X0U], PDB-6L81 [https://www.rcsb.org/structure/6L81], PDB-6M33 [https://www.rcsb.org/structure/6M33], PDB-6V6S [https://www.rcsb.org/structure/6V6S], PBD-7AS4 [https://www.rcsb.org/structure/7AS4], EMD-21074 [https://www.ebi.ac.uk/pdbe/entry/emdb/EMD-21074], EMD-21069 [https://www.ebi.ac.uk/pdbe/entry/emdb/EMD-21069], EMD-11888 [https://www.emdataresource.org/EMD-11888].

Field-specific reporting

Please select the one below that is the best fit for your research. If you are not sure, read the appropriate sections before making your selection.

- [x] Life sciences
- [ ] Behavioural & social sciences
- [ ] Ecological, evolutionary & environmental sciences

For a reference copy of the document with all sections, see nature.com/documents/nr-reporting-summary-flat.pdf

Life sciences study design

All studies must disclose on these points even when the disclosure is negative.

Sample size	Collected cryo-EM data contained 2,614 images of recombinant γ-TuRCΔN56-GCP6 , 6,127 of recombinant γ-TuRC and 11,276 for γ-TuRCΔGCP2/3. Size of the data correspond to one day microscope session with parameters specified in the method section of data acquisition for γ-TuRCΔN56-GCP6 and two day microscope session for recombinant γ-TuRC and γ-TuRCΔGCP2/3. Initial number of picked particles was 448,184 for recombinant γ-TuRCΔN56-GCP6, 436,887 for recombinant γ-TuRC and 1.89 million for γ-TuRCΔGCP2/3 . After several consecutive rounds of 3D classification, we reached final dataset of 41,054 for recombinant γ-TuRCΔN56-GCP6, 159,770 particles for recombinant γ-TuRC and 9,192 for γ-TuRCΔGCP2/3 that were used for reconstructions of γ-TuRC assembly intermediates. The final reconstructions reached resolution that was estimated by Gold Standard FSC method and sufficient for assignment of proteins.<br>For negative stain EM data sample size was adapted to acheive enough particles for sufficient 2D class avereaging. 876 images for wild-type γ-TuRC were acquired, 1,197,806 particles were picked initially. For γ-TuRCΔN56 267 images were acquired, 194,874 particles were picked initially. For γ-TuSC 530 images were acquired, 268,126 particles were picked initially. For subcomplex obtained after omitting GCP2 and GCP3 from the expression system 329 images were acquired, 183,636 particles were picked initially. For the γ-TuSC dilution series, 100 images were acquired for each dilution of the γ-TuSC dilution series and processed:1:2 dilution: 134,246 particles picked initially. 1:5 dilution: 122,712 particles picked initially. 1:10 dilution: 110,642 particles picked initially.1:10. 1:20 dilution: 76,325 particles picked initially. 1:50 dilution: 65,732 particles picked initially. 1:100 dilution: 51,950 particles picked initially. For ΔN56-GCP6-FLAG γ-TuRC from HEK T293 cells, 110,331 particles were picked on 319 micrographs. For in vivo and light microscopy experiments no statistical method was used to predict the sample size. For each technical replicate at least 10 images were taken, each containing at least 5 cells to acheive sufficient number of cells/ centrosomes/microtubules to analyze. Live cell imaging experiment was conducted by imaging at least 20 cells per condition.For batch in vitro microtubule nucelation assays no statistical method was used to predict the sample size. A wide range of conditions (concentrations) was chosen to compare the samples in a sufficient number of replicates to acount for technical fluctuations.
Data exclusions	All collected negative stain EM and cryo-EM images were used for particle picking. Cryo-EM particle selection was performed in several 3D classification rounds, and criterion was based on the quality of resulting 3D maps. This is a standard image processing practice in the cryo-EM. Negative stain EM particle selection was performed in several 2D classification rounds and criterion was based on the shape of the class averages.
Replication	Negative stain EM data acquisition and SDS page analysis was performed once for each dataset (protein purifications) with 0 repetitions. Cryo-EM dataset was acquired on one grid for each dataset with 0 repetitions. Data of batch in vitro microtubule nucelation measuremts are from four technical replicates and in vitro microtubule nucleation assay via microtubule counting from 3 technical replicates. For in vivo

experiments at least two independent experiments containing at least 3 technical replicates were conducted to confirm the reproducibility of the experiment. Immunoblots, immuno-fluorescence, live-cell imaging were repeated at least two times. IP experiments were repeated n=3 times. Protein expression and purification were done in at least 3 replicates. All independent experiments were successfully replicated.

Randomization

Positions for image acquisition in cryo-EM and Negative stain EM were selected based on the presence of the particles. Positions for image acquisition in flourescence microscopy experiments were selected on the presence of cells/microtubules. A wide field of view was used to image a suffienct number of cells/microtubles per image to avoid biased image seclection. Other experiments were not related to randomization.

Blinding

Cryo-EM, negative stain EM analysis were not blinded because it is performed computationally.For other experiments like cloning, expression protein purification it was technically not possible and for immuno-fluorescence experiments it was not feasible to apply blinding experiments because of large sample size. In other cases, the findings were confirmed by independent approaches e.g. in vitro microtuble nucelation assays.

Reporting for specific materials, systems and methods

We require information from authors about some types of materials, experimental systems and methods used in many studies. Here, indicate whether each material, system or method listed is relevant to your study. If you are not sure if a list item applies to your research, read the appropriate section before selecting a response.

Materials & experimental systems

n/a	Involved in the study
☐	☒ Antibodies
☐	☒ Eukaryotic cell lines
☒	☐ Palaeontology and archaeology
☒	☐ Animals and other organisms
☒	☐ Human research participants
☒	☐ Clinical data
☒	☐ Dual use research of concern

Methods

n/a	Involved in the study
☒	☐ ChIP-seq
☒	☐ Flow cytometry
☒	☐ MRI-based neuroimaging

Antibodies

Antibodies used

Antibodies against: GAPDH (rabbit, 1:1000, CellSignalling 14C10), Vinculin (mouse, 1:2000, Proteintech 66305-1-Ig), γ-tubulin (ms, 1:1000, Abcam Ab27074), γ-tubulin (guinea-pig, 1:50, home made (see Atorino et al. 2020)), GCP6 (rabbit, 1:1000, Bethyl A302-662A),TUBGCP3 (rabbit, 1:1000, Proteintech 15719-1-AP), GCP4 (rabbit, 1:1000, see Liu et al 2019), DYKDDDDK (FLAG-tag) (mouse, 1:1600, Cell signaling 9A3), α-tubulin (mouse, 1:500, SigmaAldrich DM1A), BubR1 (mouse, 1:200, Abcam Ab4637), Pericentrin (rabbit, 1:2000, Abcam Ab4448), penta-HIS-HRP (mouse, 1:2000, Proteintech HRP-66005), penta-His (mouse, 1:2000, QIAGEN, 34660), DDDDK tag (FLAG)(rabbit, 1:1000, Proteintech, 20543-1-AP), β-Actin (Mouse, 1:1000, Proteintech 66009-1-lg), α-tubulin (rabbit, 1:1000, MBL PM054).

Validation

anti-GAPDH (https://www.cellsignal.de/products/primary-antibodies/gapdh-14c10-rabbit-mab/2118), anti-Vinculin (https://www.ptglab.com/products/Vinculin-Antibody-66305-1-Ig.htm), anti-γ-tubulin (mouse, 1:1000, abcam Ab27074)(https://www.abcam.com/gamma-tubulin-antibody-tu-30-ab27074.html), anti-GCP6 (https://www.bethyl.com/product/A302-662A/GCP6+Antibody) and in ΔN-GCP6 knock out cell lines immunoblots, anti-DYKDDDDK (FLAG-tag) (https://en.cellsignal.de/products/primary-antibodies/dykddddk-tag-9a3-mouse-mab-binds-to-same-epitope-as-sigma-s-anti-flag-m2-antibody/8146),anti-DDDDK tag (FLAG)(https://www.ptglab.com/products/Flag-Tag-Antibody-20543-1-AP.htm), anti-α-tubulin (https://www.sigmaaldrich.com/catalog/product/sigma/t9026?lang=de&region=DE) , anti-BubR1 (https://www.abcam.com/bubr1-antibody-ab70544.html), anti-pericentrin (https://www.abcam.com/pericentrin-antibody-centrosome-marker-ab4448.html), anti-penta-His (https://www.biocompare.com/9776-Antibodies/5186822-Penta-183-His-Antibody-BSA-free-100-181-g/),anti-penta-HIS-HRP (https://www.ptglab.com/products/6-His,-His-Tag-Antibody-HRP-66005.htm), anti-GCP4 (https://www.nature.com/articles/s41586-019-1896-6), anti-TUBGCP3 (https://www.ptglab.com/products/TUBGCP3-Antibody-15719-1-AP.htm),anti-α-tubulin(rabbit, https://www.mblbio.com/bio/g/dtl/A/?pcd=PM054), anti-β-Actin (https://www.ptglab.com/products/Pan-Actin-Antibody-66009-1-Ig.htm).

Eukaryotic cell lines

Policy information about cell lines

Cell line source(s)

SF21 insect cell line obtained was obtained from EMBL protein expression facility.
RPE1 hTERT TRE3GV and HEK T293 TRE3GV cell lines were described in Atorino, E.S. et al. Nat Commun 11, 903 (2020).

Authentication

Cell lines were examined for their morphology by microscopy. RPE1 hTERT ΔN-GCP6 knock out cell lines were tested by sequencing, immuno-blot and immuno-fluorescence. HEK T293 TRE3GV ΔN56-GCP6-FLAG were tested by immuno-blot and immuno-fluorescence. RPE1 hTERT wt TUBG1mRuby2-mNeonGreenLMNB1 and RPE1 hTERT ΔN-GCP6 knock out TUBG1mRuby2-mNeonGreenLMNB1 cell lines were confirmed by fluorescence microscopy for the TUBG1mRuby2 and mNeonGreenLMNB1 integration.

Mycoplasma contamination	Mycoplasma test was for all (RPE1 hTERT TRE3GV and HEK T293 TRE3GV) cell lines and their derivated cell strains negative.
Commonly misidentified lines (See ICLAC register)	No misidentified cell lines in this study.
